# Drug screening at single-organoid resolution via bioprinting and interferometry

**DOI:** 10.1038/s41467-023-38832-8

**Published:** 2023-06-06

**Authors:** Peyton J. Tebon, Bowen Wang, Alexander L. Markowitz, Ardalan Davarifar, Brandon L. Tsai, Patrycja Krawczuk, Alfredo E. Gonzalez, Sara Sartini, Graeme F. Murray, Huyen Thi Lam Nguyen, Nasrin Tavanaie, Thang L. Nguyen, Paul C. Boutros, Michael A. Teitell, Alice Soragni

**Affiliations:** 1 Department of Orthopaedic Surgery, David Geffen School of Medicine, University of California Los Angeles, Los Angeles, CA USA; 2 Department of Bioengineering, University of California Los Angeles, Los Angeles, CA USA; 3 Jonsson Comprehensive Cancer Center, University of California Los Angeles, Los Angeles, CA USA; 4 Department of Pathology, David Geffen School of Medicine, University of California Los Angeles, Los Angeles, CA USA; 5 Department of Human Genetics, University of California Los Angeles, Los Angeles, CA USA; 6 Institute for Precision Health, University of California Los Angeles, Los Angeles, CA USA; 7 Information Sciences Institute, University of Southern California, Marina Del Rey, CA USA; 8 Department of Molecular and Medical Pharmacology, University of California Los Angeles, Los Angeles, CA USA; 9 Department of Physics, Virginia Commonwealth University, Richmond, VA USA; 10 Molecular Biology Institute, University of California Los Angeles, Los Angeles, CA USA; 11 Eli and Edythe Broad Center of Regenerative Medicine and Stem Cell Research, University of California Los Angeles, Los Angeles, CA USA; 12 California NanoSystems Institute, University of California Los Angeles, Los Angeles, CA USA; 13 Department of Urology, University of California Los Angeles, Los Angeles, CA USA; 14 Department of Pediatrics, David Geffen School of Medicine, University of California Los Angeles, Los Angeles, CA USA

**Keywords:** Imaging and sensing, Cancer models, Tissue engineering

## Abstract

High throughput drug screening is an established approach to investigate tumor biology and identify therapeutic leads. Traditional platforms use two-dimensional cultures which do not accurately reflect the biology of human tumors. More clinically relevant model systems such as three-dimensional tumor organoids can be difficult to scale and screen. Manually seeded organoids coupled to destructive endpoint assays allow for the characterization of treatment response, but do not capture transitory changes and intra-sample heterogeneity underlying clinically observed resistance to therapy. We present a pipeline to generate bioprinted tumor organoids linked to label-free, time-resolved imaging via high-speed live cell interferometry (HSLCI) and machine learning-based quantitation of individual organoids. Bioprinting cells gives rise to 3D structures with unaltered tumor histology and gene expression profiles. HSLCI imaging in tandem with machine learning-based segmentation and classification tools enables accurate, label-free parallel mass measurements for thousands of organoids. We demonstrate that this strategy identifies organoids transiently or persistently sensitive or resistant to specific therapies, information that could be used to guide rapid therapy selection.

## Abstract (web summary)

Traditional 2D cell culture platforms do not accurately reflect the physiology of human tumors. Here, authors combine bioprinting and high-speed live cell interferometry with machine learning to measure drug sensitivity at single-organoid resolution in a label-free manner.

## Introduction

Functional approaches in precision oncology entail exposing tumor cells to therapeutic interventions to identify drug candidates and to rapidly assess the potential efficacy of drugs for individual patients and therapy selection^1–8^. While a small number of actionable mutations are known, the majority of newly-diagnosed tumors lack any currently actionable genomic alteration^9^. By directly measuring the effect of drugs on tissues or cells, functional precision medicine methods can inform on the therapeutic resistance and sensitivity landscape of tumors without requiring full knowledge of the underlying molecular vulnerabilities a priori^2–4,10–17^.

The broadly adopted model systems for screening assays each have limitations. Two-dimensional (2D) cell lines are relatively simple and inexpensive to culture but fail to represent the architecture, behavior, and drug response of native tissue^18–21^. Mouse models have additional complexity but carry inherent, species-specific variations that limit their translation to human patients^22,23^. Patient-derived xenograft (PDX) models aim to better recapitulate human cancers yet are still constrained by the large cost and time associated with their use which translates to the impracticality of performing large drug screenings^1,24,25^. Three-dimensional (3D) tumor organoids are promising models for precision medicine that can be established rapidly and effectively from a variety of cell lines^26,27^ and tissue sources^28,29^, and accurately mimic a patient’s response to therapy^3,4,8,12–15,28–32^. They are physiologically-relevant, personalized cancer models well-suited for drug development and clinical applications^8,28,29,31^. We previously developed a screening platform that takes advantage of patient-derived tumor organoids seeded in a mini-ring format to automate high throughput drug testing, with results available within one week from surgery^3,16,33^. The key outstanding limitations to the broad adoption of organoid-based screenings remain the time-intensive and operator-to-operator susceptibility of the cell seeding steps as well as destructive, population-level approaches required for subsequent organoid analysis^34,35^.

To overcome these limitations, we developed an organoid screening approach that combines automated cell seeding via bioprinting with high-speed live cell interferometry (HSLCI) for non-invasive, label-free, time-resolved imaging. Bioprinting, a technique for precise, reproducible deposition of cells in bioinks onto solid supports, is rapidly gaining traction in cancer biology^36–40^. Within the deposited bioink, embedded cells can interact with physiological microenvironment components^34^. We then use HSLCI, a type of quantitative phase imaging (QPI)^41–45^, to rapidly monitor changes in dry biomass and biomass distribution of single organoids over time. The HSLCI measures the phase shift of light transmitted through the sample which is used to calculate the mass density of each organoid^46,47^. Biomass is an important metric of organoid fitness as its dynamics are the direct result of biosynthetic and degradative processes within cells^46,47^. HSLCI uses a wavefront sensing camera to enable reconstruction of the phase shift of light as it passes through a cell and interacts with matter^43,46,47^. Due to the defined linear relationship between the mass density and refractive index of biomolecules in solution, which is invariant with respect to changes in cellular content^48–52^, measured phase shifts can be integrated across the area of an image and multiplied by a conversion factor to obtain the dry biomass density of imaged cells^46,47^. This conversion factor is the inverse of the specific refractive increment, defined as the slope of the relationship between the refractive index and mass concentration of a solution^46,47,50^. QPI measurements of biomass changes allowed resolution of drug-resistant and drug-sensitive cells in 2D cell culture models within hours of treatment^41,42,45,53–57^. HSLCI-measured response profiles matched drug sensitivity from PDX mouse models of breast cancer^42^. However, HSLCI has so far been applied exclusively to screening of cancer cell lines grown in 2D or single-cell suspensions of excised PDX tumors^41,42,44,45^.

Here, we introduce a comprehensive pipeline that combines bioprinting, HSLCI, and machine learning-based organoid tracking and classification. Using cell lines as a model for 3D tumor cell growth, we demonstrate that bioprinted cells deposited in uniform, flat layers of extracellular matrix allow label-free, time-resolved, non-destructive quantification of growth patterns and drug responses at single-organoid resolution.

## Results

### Bioprinting enables seeding cells in Matrigel in suitable geometries for quantitative imaging applications

**Fig. 1:**
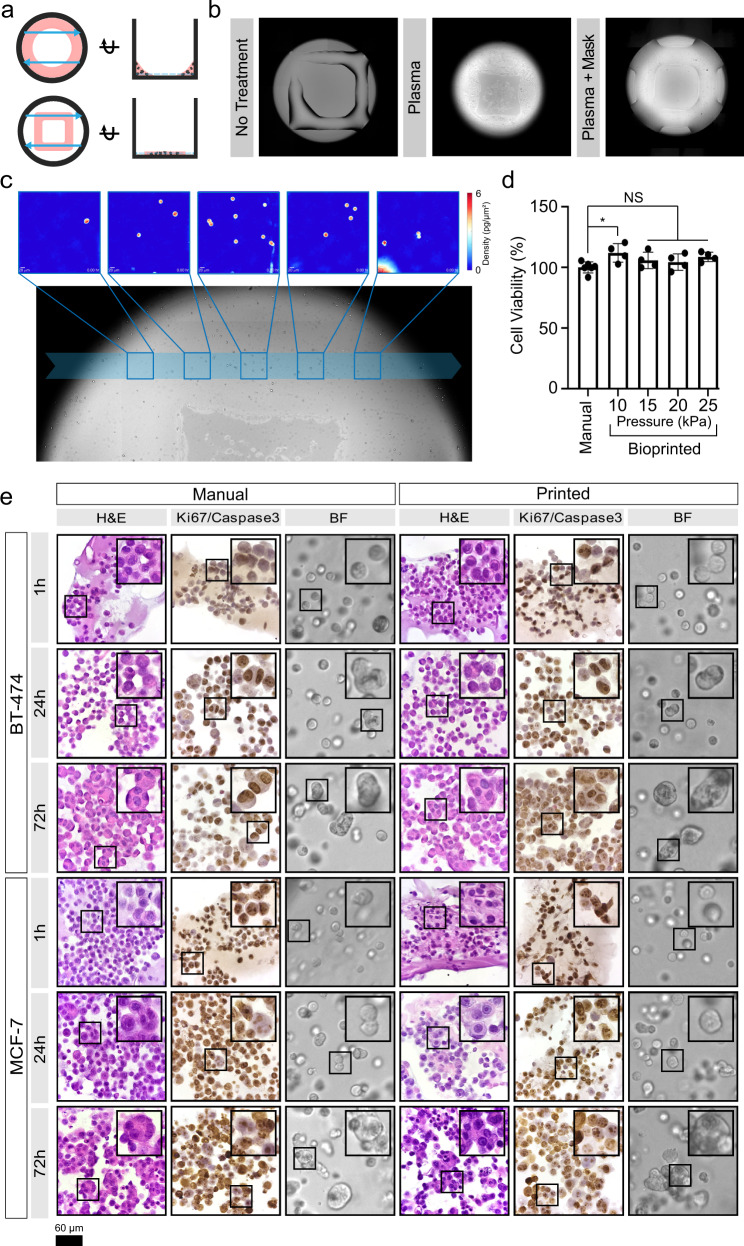
Bioprinting enables the seeding of Matrigel-encapsulated cells optimized for efficient HSLCI. **a** Schematic of wells with mini-rings (top) and mini-squares (bottom) relative to HSLCI imaging path (blue arrows). The top views (left) demonstrate that transitioning from rings to squares increases the area of material in the HSLCI imaging path. The side views (right) show that organoids in the square geometry align to a single focal plane better than organoids in a ring. **b** Plasma treatment of the well plate prior to printing optimizes hydrogel construct geometry. Bioprinting Matrigel onto untreated glass (left) generates thick ( ~ 200 µm) constructs that decreases the efficiency of organoid tracking by increasing the number of clusters out of the focal plane. Whole well plasma treatment (middle) increases the hydrophilicity of all well surfaces causing the Matrigel to spread thin ( ~ 50 µm) over the surface; however, the increased hydrophilicity also draws bioink up the walls of the well. Plasma treatment with a well mask facilitates the selective treatment of a desired region of the well (right). This leads to optimal constructs with a uniform thickness of approximately 75 µm across the imaging path. **c** Individual organoids can be tracked over time across imaging modalities. Five representative HSLCI images are traced to the imaging path across a brightfield image. **d** Cell viability of printed versus manually seeded MCF-7 cells in a Matrigel-based bioink, 1 h after plating. Data are presented as mean values ± SD. A one-way ANOVA was performed (*n* = 4, *p* = 0.0605) with post-hoc Bonferroni’s multiple comparisons test to compare all bioprinted conditions against the manually seeded control. Adjusted *p*-values were 0.0253, 0.6087, >0.9999, 0.1499 for print pressures 10, 15, 20, 25 kPa, respectively. **e** H&E staining shows the development of multicellular organoids over time regardless of seeding method. The prevalence and size of multicellular structures increases with culture time. Ki-67/Caspase-3 staining demonstrates that most cells remain in a proliferative state throughout culture time. While some apoptotic cells were observed in organoids cultured for 72 hours, the majority of cells show strong Ki-67 positivity. Ki-67 is stained brown, and Caspase-3 is stained pink. Scale bar is 60 µm. Source data is provided as a Source Data file.

To address current limitations^3,16,33^ and facilitate non-invasive, label-free imaging of 3D organoids by HSLCI, we created an automated cell printing protocol using an extrusion bioprinter. As a base, we used an organoid screening platform in which organoids are grown from cells seeded in mini-rings of Matrigel around the rim of 96-well plates, with the empty center allowing the use of automated liquid handlers, facilitating media exchanges and addition of perturbagens^3,16,32,33^. We retained the empty center architecture but altered the geometry to bioprint mini-squares of cells in Matrigel (Fig. 1a). Positioning the sides of the square in the HSLCI imaging path allows sampling of a larger area and limits imaging artifacts caused by uneven illumination at well edges^43^ (Fig. 1a). Our bioprinting protocol entails suspending cells in a bioink consisting of a 3:4 ratio of medium to Matrigel. We then transferred this material to a print cartridge, incubated at 17 °C for 30 minutes, and bioprinted into each well at a pressure between 7 and 15 kPa, resulting in <200 µm prints on standard glass-bottom plates (Fig. 1b).

We next coupled bioprinted cell lines grown in 3D clusters to an HSLCI platform. HSLCI uses a wavefront sensing camera and a dynamic focus stabilization system to perform continuous, high throughput, label-free, QPI of biological samples, tracking their biomass changes over time^41,42^. However, efficient high throughput QPI of 3D organoids using HSLCI is hindered by geometry considerations; when an object of interest is out of focus, measured phase shifts cannot be assumed to maintain a direct relationship with mass density^43^. Thus, we attempted to generate thinner layers of Matrigel to yield a relatively greater number of organoids in focus that can be quantitatively assessed at any given time. To generate thinner (<100 µm) constructs amenable to efficient, label-free HSLCI imaging, we increased the hydrophilicity of the surface of 96-well glass-bottom plates by oxygen plasma treatment^58^. We developed 3D masks composed of BioMed Amber Resin (FormLabs) to selectively functionalize the region of interest (Supplementary Fig. 1A–C). Bioprinting post-plasma treatment generated uniform mini-squares with organoids closely aligned on a single focal plane at ~70 µm thickness (Fig. 1b). The printed structures are consistent (Supplementary Fig. 1D) and amenable to massively parallel QPI by HSLCI as we aligned the legs of the bioprinted mini-square construct with the HSLCI imaging path (Fig. 1c).

Lastly, we verified that the printing parameters used did not alter cell viability by directly comparing MCF-7 cells manually seeded according to our established protocol^3,16,33^ to cells printed through a 25 gauge needle (260 µm inner diameter) using extrusion pressures ranging from 10 to 25 kPa. We did not observe any reduction in cell viability as measured by ATP release assay (Fig. 1d). These results are consistent with the existing literature as reductions in cell viability are often associated with higher print pressures (50–300 kPa)^59,60^. Taken together, this describes a method for bioprinting layers suitable for HSLCI imaging without impacting cell viability, while supporting automated liquid-handling for high throughput applications^3,16,33,61,62^.

### Bioprinted tumor cells maintain histological features of manually seeded 3D cultures

To verify that bioprinting did not perturb tumor biology, we directly compared the histology and immunohistochemical profiles of bioprinted and hand-seeded cells from two breast cancer cell lines, BT-474 and MCF-7. These lines were selected for their differing molecular features such as their human epidermal growth factor receptor 2 (HER2) and estrogen receptor (ER) status^63,64^. We grew organoids from cells seeded in maxi-rings (1 × 10^5^ cells/ring) to obtain sufficient material for downstream characterization. Cells were either manually seeded into 24 well plates^3,16,33^ or bioprinted into 8-well plates at an extrusion pressure of 15 kPa. The bioprinted cells and cell clusters were morphologically indistinguishable from manually seeded ones in brightfield images and hematoxylin and eosin (H&E)-stained sections taken 1, 24, and 72 hours after cell seeding (Fig. 1e). Both bioprinted and manually seeded cell clusters grew in size over time and bioprinting did not alter their proliferation rates (Ki-67 staining) or apoptosis (cleaved caspase-3; Fig. 1e). Hormone receptor status was in agreement with literature reports for both cell types^64–67^ and unaltered between bioprinted and manually seeded cells as shown by immunohistochemistry (IHC) for HER2 and ER (Supplementary Fig. 2). Chaperone protein expression also remained consistent with manually printed cells (Supplementary Fig. 3). Thus, bioprinting did not influence histology for the tested cell types.

### Bioprinted and manually seeded cells are molecularly indistinguishable

**Fig. 2:**
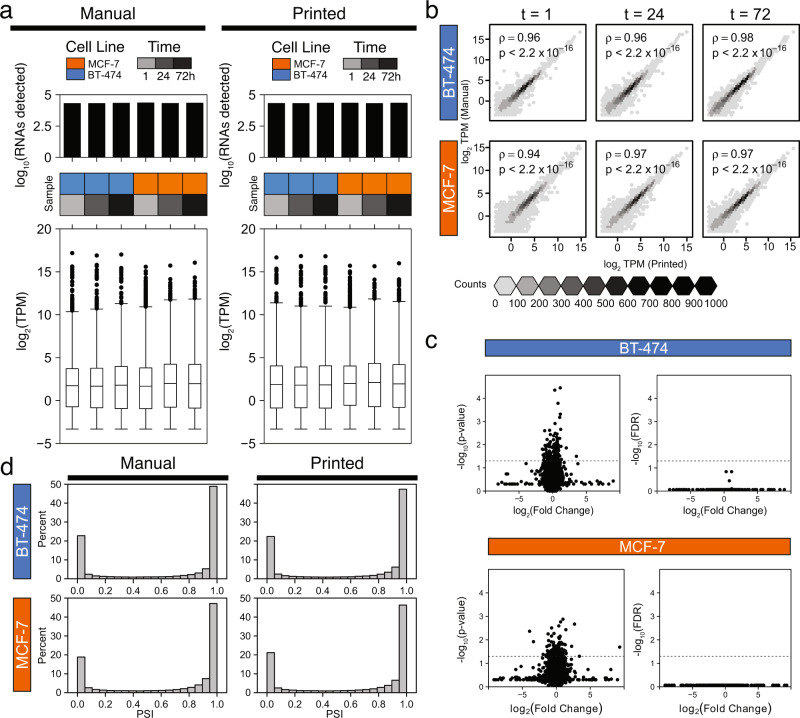
Bioprinting does not significantly alter transcriptomes. **a** Distributions of total number of RNAs detected (above) and RNA abundance (below) measured as transcripts per million (TPM) are similar between manually seeded (left) and bioprinted (right) cells. *n* = 30,544 transcripts were assessed across two independent experiments. Boxplot centers represent each median, edges of the boxes represent the 25th and 75th quartiles, whiskers represent the range of the data, and individually plotted points are outliers greater than 1.5 times the interquartile range from the median. **b** Spearman’s rank correlation of RNA abundance of manually seeded and bioprinted cell line 3D models at three time points (t = 1, 24, and 72 hours post-seeding). We found strong correlations between RNA abundance in manually seeded and bioprinted cells for both cell lines. *p*-values are derived from a two-tailed test of correlation between paired samples. **c** Volcano plots of paired, two-tailed t-test results comparing the RNA abundance of manually seeded and bioprinted MCF-7 and BT-474 with unadjusted *p*-values (left) and false discovery rate (FDR) adjusted *p*-values (right). No transcripts were preferentially expressed based upon the seeding method for either cell line (*n* = 0 out of 30,544 genes, FDR < 0.05, t-test). **d** Distribution of percent spliced in (PSI) exons are similarly distributed among BT-474 (top) and MCF-7 (bottom). The distribution of PSI is similar between manually seeded (left) and bioprinted (right) cells. PSI of 1 indicates that the exon is exclusively included, while a PSI of 0 indicates that the exon is exclusively excluded. Source data is provided as a Source Data file.

While bioprinted cells are histologically indistinguishable from manually seeded ones, this does not preclude molecular changes caused by the printing process. We therefore performed a detailed analysis of the transcriptomes of manually seeded and bioprinted cells 1, 24 and 72 hours post-seeding. By pooling RNA sequencing (RNAseq) data across two independent experiments, we assessed the distributions of 30,544 RNA transcripts and found no significant differences between seeding approaches (Fig. 2a). The overall transcriptomes of manually seeded and bioprinted cells were highly correlated (Fig. 2b), with no individual transcripts differing significantly in abundance for either cell line (FDR < 0.05, *t*-test; Fig. 2c).

We next examined pre-mRNA alternative splicing events since these can induce functional changes even in the absence of variations in mRNA levels^68–70^. We found that the density of exon-inclusion and exon-skipping isoforms was unchanged (Fig. 2d). Similarly, we found no association between the seeding method and the number of fusion transcripts (*p* = 0.0519, Mann-Whitney U-test), although a fraction of samples had large numbers of RNA fusions, reflecting the wide-spread trans-splicing and genomic instability of immortalized cell lines^71^ (Supplementary Fig. 4A). Finally, there were no significant differences in the number or nature of RNA editing sites between printed and manually seeded samples (*p* = 0.977, Mann-Whitney U-test; Supplementary Fig. 4B). These findings demonstrate that the bioprinting protocol does not significantly impact the molecular characteristics of tumor cells as measured by bulk RNA sequencing.

### Machine learning-based image segmentation and classification enables single-organoid analysis

**Fig. 3:**
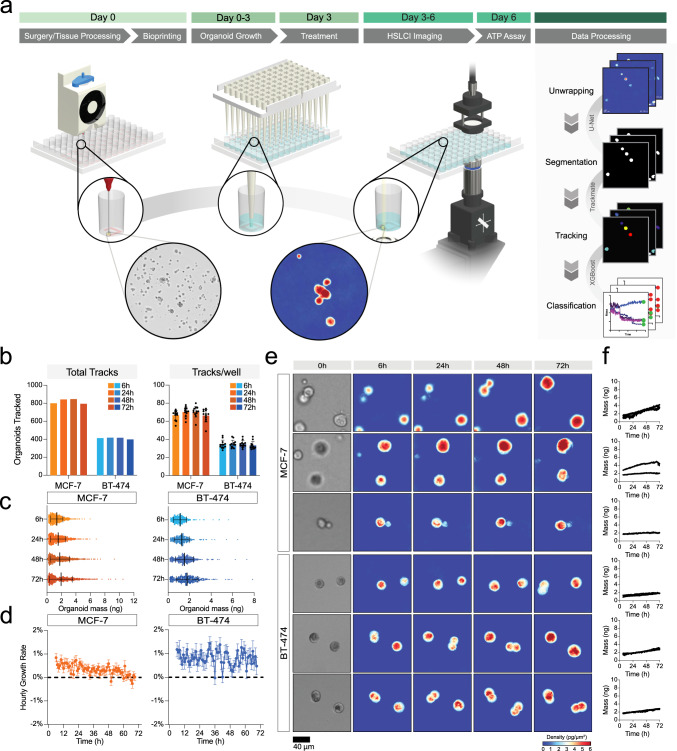
Bioprinting enables single-organoid tracking with high-speed live cell interferometry. **a** General schematic of the pipeline. Extrusion-based bioprinting is used to deposit single-layer Matrigel constructs into a 96-well plate (Day 0). Organoid model establishment and growth (Day 0–3) can be monitored through brightfield imaging. After treatment (Day 3), the well plate is transferred to the high-speed live cell interferometer for phase imaging (Day 3–6). Coherent light illuminates the bioprinted construct and a phase image is obtained. Organoids are tracked up to three days using the HSLCI and changes in organoid mass are measured to observe response to treatment. **b** Total number of tracks (left) and mean number ± SD of tracks per well (right) for cell clusters from each cell line at four time points (t = 6, 24, 48, and 72 hours after treatment). The total number of tracks across replicate wells treated with vehicle (1% DMSO) was 921 for MCF-7 (*n* = 12 wells) and 438 for BT-474 (*n* = 12 wells) **c** Mass distribution at four time points (t = 6, 24, 48, and 72 hours after treatment). Black bars represent the mean with error bars representing the standard deviation. Mean and standard deviation calculated based on *n* = 804, 855, 859, 803 for MCF-7 and *n* = 421, 420, 423, 402 for BT-474 cell clusters at t = 6, 24, 48, and 72 hours, respectively. **d** Hourly growth rate (percent mass change per hour) of tracked MCF-7 (left) and BT-474 (right) cultured in 1% DMSO. Data presented as mean ± SEM for each hour calculated based on growth rate data for *n* = 921 MCF-7 and *n* = 438 BT-474 tracked clusters. **e** Representative HSLCI-acquired phase images at four time points (t = 6, 24, 48, and 72 hours after treatment). Brightfield images taken immediately before treatment are shown on the left. **f** Calculated mass of each representative organoid over time. Source data is provided as a Source Data file.

Our complete pipeline includes cell bioprinting (day 0), organoid establishment (day 0-3), and full media replacement (day 3, Fig. 3a) followed by transfer to the HSLCI incubator. Within 6 hours of media exchange, we started to continuously image the plates through 72 hours post-treatment. At the end of the imaging period, we performed an endpoint ATP release assay to assess cell viability (Fig. 3a). For downstream data processing, we first converted interferograms collected by HSLCI to phase shift maps using the SID4 software development kit (GPU version, v741)^43^. Then we performed image segmentation and single-organoid level analyses using two types of machine learning algorithms (Fig. 3a).

To reliably identify unique cell clusters within each imaging frame despite the presence of background noise, debris, and out-of-focus organoids, we performed image segmentation using a U-Net architecture^72^ with ResNet-34^16,73^ as the backbone. U-Net, a type of convolutional neural network (CNN), consists of an encoder that extracts rich feature maps from an input image and a decoder that expands the resolution of the feature maps back to the image’s original size. The long skip connections between the encoder and decoder propagate pixel-level contextual information into the segmented organoid masks. The resulting segmentation images are very detailed even when provided small training datasets. We used a training dataset consisting of manually labeled organoids in 100 randomly selected imaging frames. This model created binary masks indicating whether each pixel of the image belonged to an organoid or the background with a mean Jaccard Index of 0.897 ± 0.109 at the 95% confidence level. The CNN reliably created masks omitting phase artifacts resulting from aberrant background or out-of-focus organoids (Supplementary Fig. 5).

Next, we determined the mass of each cluster in segmented masks by integrating the phase shift over the organoid area and multiplying by the experimentally determined specific refractive increment^48,50,51,54,74^. We assembled data from discrete segmented cell clusters and organoids into time-coherent tracks using TrackMate^75,76^ and filtered mass versus time data for each tracked object using a XGBoost classifier^77^ trained to exclude organoids moving in and out of focus, frequently overlapping and/or separating from neighboring clusters, or incorporating debris (Supplementary Table 1). We validated the model via cross-validation with 3-fold resampling of the sample population. The 3-fold resampling cross-validation score of the classifier was 93.1 ± 1.3% with a 0.966 ± 0.020 AUC (Supplementary Table 2). We also observed trends in the features used to classify each track, with excluded tracks typically having an increased number of missing frames as well as smaller interquartile ranges, and smaller initial mass (Supplementary Figure 6).

### Trends in mass accumulation can be quantified by HSLCI with single-organoid resolution

HSLCI-based imaging allowed continuous tracking of *n* = 921 MCF-7 cell line-derived organoids in 12 replicate wells (median: 78.5/well) and *n* = 438 BT-474 in 12 replicate wells (median: 36/well, Fig. 3b). Due to cells moving in and out of the field-of-view (FOV), the number of tracked objects at each time point varied slightly. Overall, we tracked an average of 821 ± 28 MCF-7 and 412 ± 9 BT-474 cell clusters at any given time throughout imaging (Fig. 3b).

Unlike chemical endpoint assays or other live imaging modalities, HSLCI-based imaging facilitates parallel mass measurements of individual cells and organoids. The initial average measured mass at the start of imaging, corresponding to approximately 78 h total in culture, was larger for MCF-7 (1.36 ± 0.84 ng) than BT-474 (1.12 ± 0.61 ng, Fig. 3c). Considering a single cell mass of 200–300 pg^55^, clusters contained ~4–7 cells after 3 days in culture as measured by HSLCI, consistent with the observed histopathology (Fig. 1e). The difference in size between MCF-7 and BT-474 persisted throughout the entire imaging duration (Supplementary Table 3). BT-474 grew at a rate of 0.80 ± 6.07% per hour while MCF-7 demonstrated slower average hourly growth rates (0.33 ± 4.94% per hour, Fig. 3d). The growth rate of the BT-474 in 3D was slightly slower than BT-474 cells observed after 6 hours in 2D culture ( ~ 1.3%), while the MCF-7 in 3D showed a much lower growth rate than previously reported 2D cultures ( ~ 1.7%)^55^. We also observed positive associations between initial mass and growth rate in organoids originating from both lines; however, the association between these factors is stronger for MCF-7 cells (Supplementary Fig. 7). The data presented provides evidence that cell-line specific 3D growth characteristics can be quantified by HSLCI.

### Drug responses in 3D cultures can be quantified by HSCLI

**Fig. 4:**
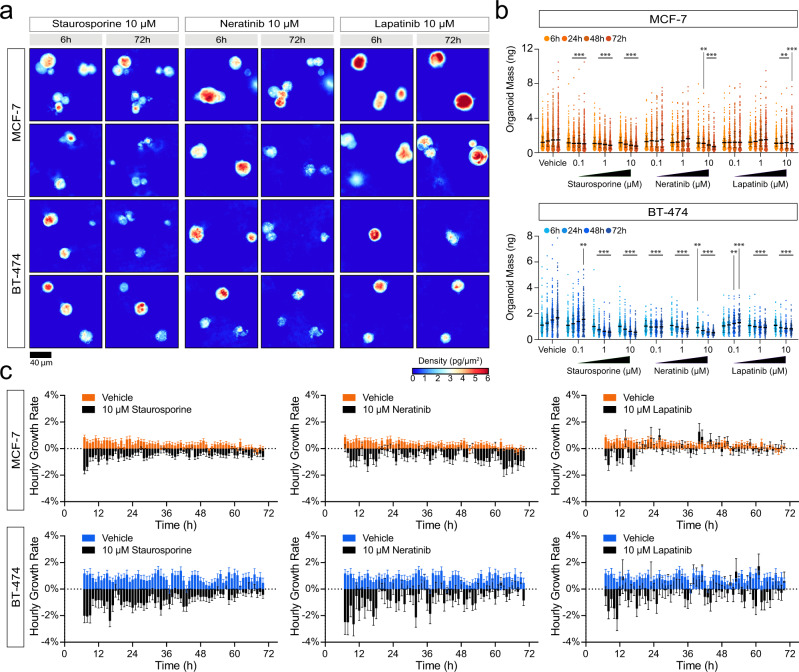
HSLCI enables high throughput, longitudinal drug response profiling of 3D models of cancer. **a** Representative HSLCI-acquired phase images of MCF-7 and BT-474 grown in 3D and treated with 10 µM staurosporine, 10 µM neratinib, and 10 µM lapatinib. **b** Each bar represents the mass distribution at 6-, 24-, 48-, and 72 hours post-treatment (left to right). Black horizontal bars represent the median with error bars representing the interquartile range of the distribution. Sample sizes, summary statistics, and exact *p*-values for each condition are available in Supplementary Table 3. Statistical significance was assessed using Kruskal-Wallis tests. For samples with *p*-values lower than 0.05, we performed two-tailed Mann-Whitney U-tests against the vehicle control at the respective time points. *p* < 0.05 is denoted by *, *p* < 0.01 is denoted by **, and *p* < 0.001 is denoted by ***. **c** Hourly growth rates, calculated as percent mass change per hour, are compared between organoids treated with 10 µM staurosporine and vehicle, 10 µM neratinib and vehicle, and 10 µM lapatinib and vehicle. Data are presented as mean growth rate ± SEM. Growth rate data is derived from *n* = 921, 592, 249, and 292 MCF-7 cell clusters and *n* = 438, 299, 110, and 127 BT-474 clusters treated with the vehicle, 10 µM staurosporine, 10 µM neratinib, and 10 µM lapatinib, respectively. Source data is provided as a Source Data file.

We then tested the utility of our platform in detecting drug responses in high throughput 3D organoid screenings (Fig. 3a). As proof-of-principle we tested staurosporine, a non-selective protein kinase inhibitor with broad cytotoxicity^78^, neratinib, an irreversible tyrosine kinase inhibitor targeting EGFR and HER2^79^, and lapatinib, a reversible tyrosine kinase inhibitor also targeting EGFR and HER2^80^. We tested staurosporine at 0.1, 1, and 10 µM, while neratinib and lapatinib were screened at 0.1, 1, 10, and 50 µM (Fig. 4 and S8). The concentration ranges tested include and extend beyond the maximum plasma concentrations reported for both lapatinib (4.2 μM)^81^ and neratinib (0.15 µM)^82^. We excluded wells treated with 50 µM neratinib from image analysis because drug precipitation at this concentration resulted in optical opacity. Precipitates and other opaque materials limit data acquisition by causing uninterpretable phase images.

Representative HSLCI images demonstrate a range of responses to treatment (Fig. 4a). Six hours post-treatment, we detected a significant decrease in the average mass of BT-474 clusters treated with 10 µM neratinib relative to the control. The average masses at the start of the imaging window (6 hours post-treatment) did not significantly differ from the vehicle control for all other conditions (Fig. 4b, Supplementary Table 3). After 24, 48, and 72 hours, we observed significant differences in a number of treated samples (Supplementary Table 3). After 24 hours, control MCF-7 cell clusters averaged 1.56 ± 1.05 ng, while those treated with 1 μM and 10 μM staurosporine showed significant reductions in average mass to 1.18 ± 0.77 ng (*p* = 1.93 × 10^−9^, Mann-Whitney U-test) and 1.11 ± 0.69 ng (*p* = 1.49 × 10^−13^, Mann-Whitney U-test), respectively. BT-474 showed a similar pattern after 24 hours with control organoids averaging masses of 1.27 ± 0.69 ng while staurosporine-treated clusters averaged 0.76 ± 0.39 ng (1 μM, *p* = 2.27 × 10^−32^ Mann-Whitney U-test) and 0.79 ± 0.44 ng (10 μM, *p* = 1.59 × 10^−28^, Mann–Whitney U-test). Both MCF-7 and BT-474 rapidly responded to treatment with 1 μM staurosporine, as visualized by plotting normalized growth curves (Supplementary Fig. 9) and by the reduction in the average growth rate (Fig. 4c).

Responses to lapatinib and neratinib reflected cell line-specific trends consistent with HER2 expression (Supplementary Fig. 2). BT-474 quickly showed sensitivity to both neratinib and lapatinib, while MCF-7 only exhibited sensitivity to 10 µM lapatinib and neratinib (Fig. 4b, Supplementary Table 3). After 24 hours, the mean mass of BT-474 clusters treated with 0.1 µM neratinib decreased to 0.97 ± 0.44 ng from 1.27 ± 0.69 ng (*p* = 4.86 × 10^−6^, Mann-Whitney U-test) and those treated with 1 µM lapatinib decreased to 1.00 ± 0.49 (*p* = 3.37 × 10^−5^, Mann–Whitney U-test). When comparing EC_50_s of 2D cultures, manually seeded, and bioprinted 3D cultures, we found no significant change, albeit we observed a trend toward lower EC_50_ for bioprinted MCF-7 treated with neratinib (Supplementary Fig. 10, Supplementary Table 4). Results were consistent across independent experiments (Supplementary Fig. 11).

### Intra-sample heterogeneity of drug responses

**Fig. 5:**
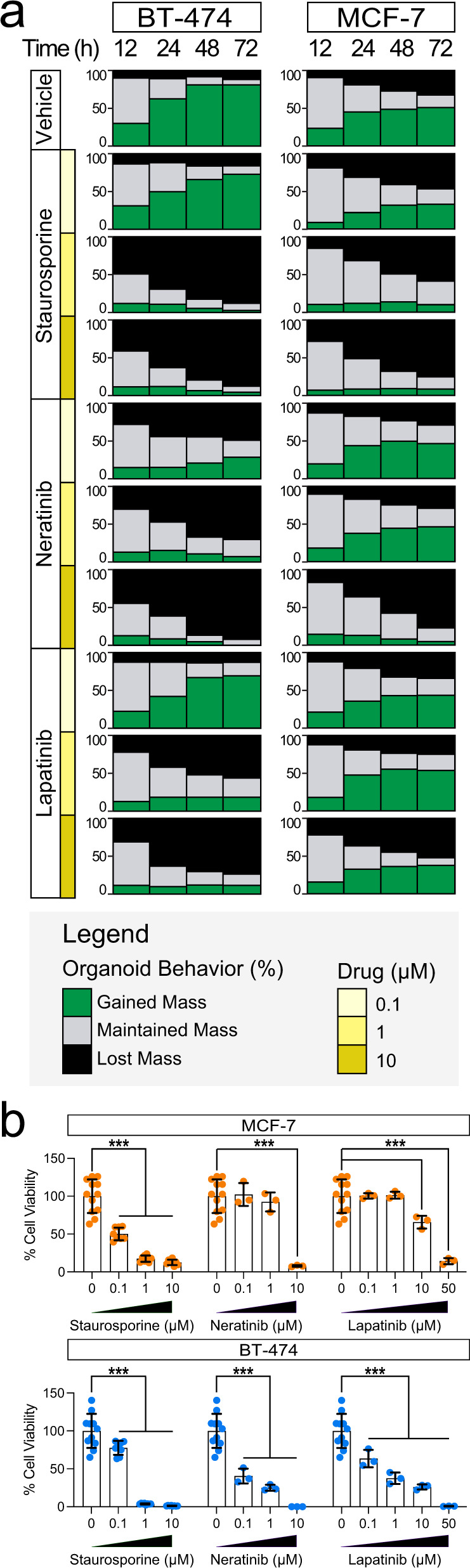
HSLCI enables identification of resistant and sensitive 3D cluster subpopulations and discerns response to treatment earlier than endpoint assays. **a** Plots showing the percentage of tracked clusters in each condition that gain (green) or lose (black) more than 10% of their initial mass 12, 24, 48, 72 hours after treatment. **b** Relative viability of treated wells in HSLCI-imaged plates of bioprinted MCF-7 and BT-474, determined by endpoint ATP release assays. Bars represent the mean and error bars represent the standard deviation. Each point represents the normalized luminescence signal from independent replicates. *n* = 12 and *n* = 11 wells were treated with the vehicle control for MCF-7 and BT-474, respectively. For treated wells in both experiments, *N* = 8 replicates were screened for each concentration of staurosporine and *N* = 3 replicate wells were screened for each concentration of both lapatinib and neratinib. Statistical significance was assessed using a two-tailed, unpaired t-test with Welch’s correction. *p* < 0.05 is denoted by *, *p* < 0.01 is denoted by **, and *p* < 0.001 is denoted by ***. Exact *p*-values are reported in Supplementary Table 6. Source data is provided as a Source Data file.

Our combination of HSLCI with machine learning-based tools provides mass tracking of individual cell clusters and organoids, allowing quantitation of intra-sample heterogeneity (Fig. 4b, Supplementary Movies 1 and 2). We assessed the ratio of imaged 3D clusters that gained, lost, and maintained mass over 12, 24, 48, and 72 hours for both control and treated samples (Fig. 5a, Supplementary Table 5). In the absence of drug treatment, 11.9% of BT-474 3D clusters lost more than 10% of their initial mass and 80.9% gained more than 10% of their initial mass over 72 hours. In contrast, only 50.8% of MCF-7 gained mass, and 32.6% lost mass. This heterogeneity in populations increases over time, with 23.2% of MCF-7 gaining more than 10% mass within 12 hours. This proportion nearly doubles to 44.8% after 24 hours but remains consistent at 48.6% and 50.8% after 48 and 72 hours, respectively. This pattern differs from BT-474 as the population of organoids that gained mass continually increases over the first 48 hours before plateauing between 48 and 72 hours. BT-474 that gained >10% mass increased from 30.1% after 12 hours, to 62.4% after 24 hours, and 80.9% after 72 hours.

Upon treatment, we observed both inter-sample (MCF-7 vs BT-474) and intra-sample heterogeneity. In the presence of the HER2-targeting lapatinib (10 µm), 37.7% of MCF-7 continued to grow and an additional 10.0% maintained their mass after 72 hours of treatment (Fig. 5a, Supplementary Table 5). When treated with 10 µM neratinib, only 4.5% of MCF-7 clusters gained mass, while 18.1% remained stable. In contrast, BT-474 showed greater sensitivity to both drugs, with 11.4% of cell clusters growing and 73.7% losing mass with 10 µM lapatinib treatment (vs 11.9% for controls), and no cell cluster growing after exposure to 10 µM neratinib for 72 hours (Fig. 5a, Supplementary Table 5). A subset of BT-474 cells showed high sensitivity to 0.1 µM of both lapatinib and neratinib. In response to lapatinib, 12.8% of BT-474 clusters lost mass, while 17.9% maintained stable mass. When treated with neratinib, 49.1% lost mass and 22.4% maintained stable mass. Both responses contrasted with organoids treated with vehicle, of which 11.9% lost and 7.2% maintained mass. The heightened sensitivity of BT-474 cells to lapatinib and neratinib is expected given the higher expression of HER2 found in these cells^63^ (Supplementary Fig. 3).

A fraction of cells in all treatment-cell combinations were unresponsive to the drugs tested (Fig. 5a, Supplementary Fig. 9, Supplementary Table 5). These grew at similar rates to their vehicle-treated counterparts and comprised between 7.8 and 87.1% of all organoids depending on the cell line and drug. For example, 37.7% of MCF-7 cell clusters treated with 10 µM lapatinib grew after 72 hours, while an additional 10% maintained stable mass (Supplementary Table 5). Similarly, when treated with 10 µM neratinib for 72 hours, nearly 8% of BT-474 maintained their mass, and when exposed to 10 µM lapatinib for 72 hours, the proportion increased to over 25% (Supplementary Table 5). Our findings are indicative of a resistant population of organoids that can be rapidly identified by HSLCI imaging. These persisters may provide a unique model for understanding de novo and acquired treatment resistance.

Lastly, to validate the responses measured by HSLCI, we performed an endpoint ATP release assay on the same plates used for HSLCI imaging and assessed viability at the end of the 72-hour treatment (Fig. 5b). The ATP assays confirmed that both cell lines are highly sensitive to staurosporine, with near-zero viability at the 1 and 10 µM concentrations. Additionally, BT-474 showed significant reductions in viability when treated with 0.1 µM lapatinib and 0.1 µM neratinib for 72 hours (Supplementary Table 6). Overall, the results of the cell viability assays after 72 hours confirmed the trends observed in as little as 6 hours by HSLCI but fail to capture intra-sample variability.

## Discussion

Cancer therapy is increasingly moving towards treatments tailored to each patient’s unique and heterogeneous disease^83,84^. Molecular precision medicine requires knowledge of the associations between molecular features and drug response;^1,85^ however, many of these relationships have yet to be established^86^. Functional precision medicine bypasses the need to have prior knowledge of these associations and several studies have related in vitro response with patient outcomes^4,12,13,32^. Key limitations towards the broad adoption of functional precision medicine have been the creation of physiological culture models, the development of high throughput systems, and the difficulty in measuring organoid heterogeneity^7,31,34,35,87^. Our pipeline overcomes each of these barriers by incorporating a robust 3D organoid bioprinting protocol and an imaging approach that facilitates single-organoid analysis of response to treatment.

We introduced bioprinting to enhance the throughput and consistency of our previously published organoid screening approaches^3,16,33^. We opted to print a Matrigel-based bioink due to its ability to preserve tumor characteristics ex vivo^3,21^. Matrigel behaves as a liquid in a narrow temperature range under its gelation temperature of 6–8 °C. Above these values, it shows time- and temperature-dependent changes in viscosity while it thermally crosslinks^88,89^. Without strict control of temperature and bioprinting preparation procedures, bioink can vary in viscosity. As a result, extrusion pressure may need to be adjusted with each print. Although these physical properties complicate its suitability for bioprinting, we show that bioprinting simple, single-layer Matrigel structures is attainable with strict temperature regulation. We further enhanced the quality of the Matrigel deposition by selectively modifying the print substrate with oxygen plasma treatment. The introduction of 3D plasma masks (Supplementary Figure 1) facilitated the selective treatment of a square region in each well. The increased hydrophilicity of the substrate in the exposed region guides the spreading of the material to maximize consistency in deposition volume and construct thickness while preventing obstruction of the center of the well. Bioprinting allowed us to finely control the size and shape of the deposited gel constructs, facilitating the use of HSLCI for downstream analysis (Fig. 1).

We have optimized a low pressure, temperature-controlled extrusion-based bioprinting protocol to avoid altering tumor cells. Though it is possible that a subset of cells may be influenced by the bioprinting process, our bulk RNAseq analysis suggests this would be limited to a very small subpopulation. Overall, the sum of our orthogonal findings supports the conclusion that the cells we tested were largely unaffected by the mild bioprinting conditions used in our studies. This applies to the specific combination of bioprinting parameters and cell models included in this work. It will be important to continue evaluating the biological effects of bioprinting in the context of future studies that either use alternative printing protocols or are focused on different patient-derived samples.

Previous studies have used interferometry to quantify the mass of individuals cells cultured on 2D substrates to study cell division^90^, cytoskeletal remodeling^91^, mechanical properties^92^, and response to treatment^41,42,54^. Tomographic QPI has also been used to obtain high-resolution images of 3D objects such as cerebral organoids^93^ and cancer organoids^94^. An enduring challenge of adapting high throughput quantitative imaging techniques to organoid models of cancer is efficient visualization of 3D clusters^35,95^. HSLCI imaging records 2D projections of the phase shift of incident light caused by cultured cells or organoids at a single focal plane relative to the plate surface^41^. 2D phase shift maps only provide accurate biomass measurements for objects in focus^43,96^. When using HSLCI to measure biomass, efficiency can be reduced by organoids moving orthogonally to the focal plane over the duration of imaging. We mitigated this challenge by using bioprinting to generate uniform, thin constructs that maximized the number of organoids that could be tracked in parallel, coupled with a machine learning-based organoid classifier that excludes out-of-focus objects from the analysis. Alternatively, an imaging system capable of capturing multiple z-planes across the plate could address this issue. Such an approach, however, would decrease the frequency of organoid imaging and increase the size of the resulting dataset, which poses different challenges for timely and efficient data analysis.

By introducing region-specific reference images and machine learning-based methods for image segmentation and track filtering, we have been able to increase the number of organoids tracked approximately 15-fold from initial analyses in which only approximately 1% of organoids analyzed were retained using standard approaches (Supplementary Fig. 11). Further improvements will include shortening the 6-hour delay between drug treatment and imaging start, which will allow us to capture highly sensitive organoids undergoing cell death within that timeframe. Lastly, due to the large amount of data generated using HSLCI (approximately 250 GB per plate/day), data analysis remains a time-limiting factor.

Our work uses bioprinting in combination with time-resolved HSLCI for high throughput, label-free biomass profiling of 3D models of cancer. While other groups have previously bioprinted organoid models^97–100^ to assess drug treatments^98–100^, most of these systems, like other screening approaches, used endpoint chemosensitivity assays that return well-level average measurements at a single time point^95^. These endpoint assays are limited in their ability to uncover transient responses and heterogeneity in growth and drug sensitivity phenotypes^95^. In contrast, the time-resolved, organoid-level information returned by HSLCI enables the characterization of rare, drug-resistant cell subpopulations, providing additional insight that cannot be resolved by population-based assays. Genomic characterization of tumors has demonstrated that these malignancies are collections of evolutionarily-related subclones, rather than homogeneous populations^101–104^. This genetic diversity is one of the several factors that contributes to differential response to treatment. Endpoint assays, such as live-dead staining or ATP release quantification, characterize the average response to treatment. Though they may be useful for identifying drug sensitivity in majority cell populations, they fail to account for the response of resistant clones that may also be present. In the clinical setting, failure to treat the resistant populations may cause recurrence and long-term disease progression after initial responses^105–107^. Despite the development of 3D cancer models with varying extents of complexity and scalability, functional screening assays have been hindered by their inability to consider this heterogeneity of tumor responses. HSLCI allows non-invasive, label-free tracking of various features of bioprinted organoids over time, including size, motility, and mass density at single-organoid resolution. Because of the ability to quantitatively measure time-resolved, individual mass changes in response to treatment, it is possible to identify and isolate responsive and resistant subpopulations of cells which can lead to more informed clinical decision making when selecting a treatment approach^45^. The combination of throughput, time resolution, and number of organoids sampled^94,108,109^ paired with our short experimental time frame from seeding to drug sensitivity results^3,16,33,110^, make our HSLCI-based method valuable for screening tumor organoid models for research and, in the future, for possible clinical applications.

## Methods

### 2D cell culture

MCF-7 and BT-474 breast adenocarcinoma cell lines were obtained from the American Type Culture Collection (ATCC). All cell lines were grown for a maximum of 10 passages in RPMI 1640 medium (Gibco 22400-089) supplemented with 10% fetal bovine serum (FBS, Gibco 16140-071) and 1% antibiotic-antimycotic (Gibco 15240-062). Both cell lines were periodically authenticated by short tandem repeat profiling using the GenePrint 10 kit (Laragen). For all 2D drug screening experiments, MCF-7 and BT-474 cells were plated manually in 96-well plates (Corning 3603) at 2500 cells/100 µL per well in RPMI 1640 medium with 10% FBS and 1% antibiotic-antimycotic, and then incubated at 37 °C and 5% CO_2_ for 3 days.

### Manually seeded 3D organoids

Organoids were seeded manually according to our previously published protocols^3,16,33^. Briefly, single cells suspended in a 3:4 mixture of Mammocult (StemCell Technologies 05620) and Matrigel (Corning 354234) were deposited around the perimeter of the wells of either 24-well or 96-well plates. The cell suspension was kept on ice throughout the seeding process to prevent the gelation of the Matrigel. To seed organoids in a 96-well plate (Corning 3603), a pipette was used to distribute 5 µL of cell suspension (5 × 10^5^ cells/mL) along the bottom perimeter of each well. Once all mini-rings were generated, plates were incubated at 37 °C and 5% CO_2_ for 20 minutes to solidify the Matrigel, and 100 µL of pre-warmed Mammocult medium was added to the center of each well using an epMotion 96 liquid handler (Eppendorf). To generate larger rings (maxi-rings) in 24-well plates (Corning 3527), 70 µL of cell suspension (1.4 × 10^6^ cells/mL) was deposited around the perimeter of each well. Following seeding, the plate was incubated at 37 °C and 5% CO_2_ for 45 minutes to solidify the Matrigel, and 1 mL of pre-warmed Mammocult was added to the center of each well. Plates were imaged daily using a Celigo S Imaging Cell Cytometer (Nexcelom) in brightfield mode.

### 3D printing plasma masks

Custom well masks were designed to meet the specifications of the well plates that were used in these experiments (Supplementary Fig. 1). The design was generated in Inventor 2020 (Autodesk) and printed using a Form3B (FormLabs) using the Biomed Amber resin (FormLabs). The design was exported as an STL file and imported into the PreForm (FormLabs) software to arrange the parts. After printing, parts were post-processed in two washes of isopropanol, air-dried for at least 30 minutes, and cured for an additional 30 minutes at 70 °C in the Form Cure (FormLabs).

### Bioprinted 3D organoids

Cells were bioprinted using a CELLINK BioX with a Temperature-Controlled Printhead. G-code files were written to print the desired single-layer geometry. MATLAB (MathWorks) was used to integrate these standardized blocks into full G-code files with the defined coordinates for each well. 8-well plates were used when printing the maxi-rings for IHC and RNAseq as the depth of the well in a standard 24-well plate prohibited the use of 0.5” length needles. For each time point, four rings with a diameter of 14.5 mm were printed for RNAseq ( ~ 2 × 10^5^ cells total), while four sets of concentric 14.5 mm, 12.5 mm, and 10.5 mm diameter rings were used for IHC analysis ( ~ 5 × 10^5^ cells total). Mini-squares with side length 3.9 mm were printed into glass-bottom plates (Cellvis P96-1.5H-N) for drug screening and HSLCI imaging. The mini-squares were inscribed within the circular well with sides parallel to the sides of the well plate. All bioprinting processes utilized the same material deposited for manually seeded organoids: a single-cell suspension in a 3:4 mixture of Mammocult and Matrigel on ice. After vortexing briefly, the mixture was transferred into a 3 mL syringe to remove air bubbles. The mixture was then transferred to a room temperature 3 mL bioprinter cartridge (CELLINK) by connecting the syringe and cartridge with a double-sided female Luer lock adapter (CELLINK). The loaded cartridge was incubated in a rotating incubator (Enviro-Genie, Scientific Industries) for 30 minutes at the print temperature.

During the incubation period, the printer was sterilized with the built-in UV irradiation function, the printhead was set to the print temperature, and the masked 96-well plates were treated with oxygen plasma. Briefly, well masks were autoclaved prior to use, inserted into the well plate, and pressed in contact with the glass surface. Masked plates were treated with oxygen plasma in a PE-25 (Plasma Etch) for 30–90 seconds, 15 minutes prior to bioprinting. After plasma treatment, the well plate was placed in the bioprinter, and Automatic Bed Levelling was performed.

Once the incubation period ended, a 0.5” 25-gauge needle (CELLINK) was attached to the cartridge which was loaded into the pre-cooled printhead. The printer was primed by extruding a small volume of material at 10–15 kPa prior to calibrating the printer. The material in the needle gelled during the printer calibration which took approximately 2 minutes. After calibration, a second extrusion using 40 kPa was performed to clear the needle of the gelled material prior to starting the print and ensure unobstructed material extrusion. To create constructs of the appropriate thicknesses, prints in 8-well plates were extruded at 15 kPa while prints in 96-well plates were extruded at 7–15 kPa. Each print of mini-squares in a 96-well plate is deposited by the bioprinter in approximately four minutes. After printing, the constructs were imaged and incubated at 37 °C for at least 30 minutes to solidify the matrix and 100 μL of Mammocult medium was then added.

### Sample preparation for RNA sequencing

Two independent experiments were conducted using an identical protocol. In each experiment, four maxi-rings were printed and manually seeded for each cell line and time point. Printed and manually seeded samples were seeded from a single bioink preparation for each cell line to preclude batch-to-batch variation from analysis. All organoids were seeded at the same time and the collection protocol was performed at 1-, 24-, and 72-hour time points, harvesting four rings each time. Organoids were released from the Matrigel in preparation for RNAseq. After media was aspirated from each ring, 1 mL of pre-chilled dispase (5 mg/mL, Life Technologies 17105-041) was added to each ring. After a 20-minute incubation at 37 °C, the cell suspension was collected and pelleted by centrifugation at 1500xg for 5 minutes and washed with 45 mL of PBS before centrifuging again at 2000xg for an additional 5 minutes. Once all liquid was aspirated, the tubes were rapidly frozen and stored at −80 °C. Frozen cell pellets ( ~ 2×10^5^ cells) were then transferred to the Technology Center for Genomics & Bioinformatics (TCGB) at UCLA for RNAseq. Sequencing was performed on a NovaSeq SP (Illumina) using the 2 ×150 bp paired-end protocol.

### Library preparation

Libraries for RNAseq were prepared with a KAPA Stranded mRNA-Seq Kit (Cat.KK8420). The workflow consisted of mRNA enrichment and fragmentation, first-strand cDNA synthesis using random priming followed by second-strand synthesis converting cDNA:RNA hybrids into double-stranded cDNAs (dscDNA), with dUTP incorporation into the second cDNA strand. cDNA generation was followed by end repair to generate blunt ends, A-tailing, adaptor ligation, and PCR amplification. Different adaptors were used for multiplexing samples in one lane. Data quality was checked using Illumina SAV. Demultiplexing was performed with the Illumina Bcl2fastq v2.19.1.403 software.

### RNA sequencing data processing and analysis

FASTQ files were processed using UCLA-CDS pipelines to align and quantify RNA-seq data. Pipeline-align-RNA v6.2.2 performs quality control with FastQC, trims reads with FASTP v0.21.0^111^, aligns with STAR v2.7.6^112^, marks duplicates with Picard tools MarkDuplicates Spark v4.1.4.1^113^, checks duplication rate with dupRadar v1.24.0^114^ and outputs sorted BAM files. Reads were aligned to human genome GRCh38.p13. Pipeline-quantitate-RNA v2.0.0 performs isoform and gene quantitation with RSEM v1.3.3^115^. Transcripts with low abundance in all samples (TPM < 0.1; transcripts per million) were excluded resulting in 30,544 transcripts included in the analysis. Pipeline-quantitate-SpliceIsoform v2.0.5 quantitates the relative usage of splice isoforms with rMATS v4.1.0^116^. Pipeline-call-RNAEditingSite v5.6.0 calls RNA editing events with REDItools2 v1.0.0^117^. Pipeline-call-FusionTranscript calls gene fusion events with FusionCatcher v1.33^118^. RNA editing sites were filtered to include adenosine to inosine events with sufficient coverage (Q30 > 10) and frequencies above 0.9. Raw and processed data is available on GEO.

RNA abundance was averaged across replicates. We used a paired t-test to perform hypothesis statistical testing on RNA abundance and Mann Whitney U-tests on the number of fusion transcripts and editing sites between bioprinted and manually seeded tumor organoids. We adjusted for multiple hypothesis testing using the false discovery rate (FDR) method, using threshold FDR < 0.05. Statistical analyses and data visualization were performed in the R statistical environment (v4.0.2) using the BPG^119^ (v6.0.1) package.

Germline variants from our samples were called using the GATK RNAseq short variant discovery (SNPs + Indels) pipeline^120^ and compared to those from the CCLE cell lines using vcfeval (rtg-tools v3.12.1)^121^ to validate sample cell line identity post-hoc.

### Immunohistochemistry

Immunohistochemical staining was performed on manually seeded and bioprinted organoids seeded in 24 or 8-well plates, respectively. A detailed procedure has been published^33^. Briefly, samples were prepared for histological analysis by carefully aspirating all media from the well without disrupting the construct and fixing in 10% buffered formalin (VWR 89370-094). The fixed organoids were harvested, transferred to a conical tube and pelleted by centrifugation at 2000xg for 5 minutes. HistoGel (Thermo Scientific HG-40000-012) was then added to the pellet. Once solidified, the cell pellet in HistoGel was placed in a histologic cassette and sent to the UCLA Translational Pathology Core Laboratory (TPCL) for dehydration and paraffin embedding.

Slides (8 µm thin sections) were baked for 20 minutes at 45 °C and de-paraffinized in xylene followed by washes in ethanol and deionized water. For H&E staining, a Hematoxylin and Eosin Stain Kit (Vector Labs H-3502) was used according to the manufacturer’s protocol. For Ki-67/Caspase-3, HER2, ER staining and chaperone staining, Peroxidazed-1 (Biocare Medical PX968M) was applied for 5 minutes at room temperature to block endogenous peroxidases. Next, antigen retrieval was performed using Diva Decloaker (Biocare Medical DV2004LX) in a 2100 Retriever (Prestige Medical) heating at 110 °C for 15 minutes. Blocking was performed at room temperature for 5 minutes with Background Punisher (Biocare Medical BP947H) for all antibodies beside Hsp27, 70 and 90 beta that were incubated for 30 minutes. Primary Ki-67/Caspase-3 staining was performed overnight with commercially available pre-diluted Ki-67/Caspase-3 (Biocare Medical PPM240DSAA) solution at 4 °C after an additional 2-minute Background Punisher treatment post-antigen retrieval, and secondary staining was performed with Mach 2 Double Stain 2 (Biocare) solution for 40 minutes at room temperature. Primary antibodies for HER2 (Novus Biologicals, CL0269) and ER (Abcam, E115) staining were diluted 1:100 in Da Vinci Green Diluent (Biocare Medical PD900L). The HER2 antibody was incubated overnight at 4 °C while the ER antibody was incubated at room temperature for 30 minutes. Secondary staining was performed with Mach 3 Mouse Probe and Mach 3 Mouse HRP-Polymer for HER2 and Mach 3 Rabbit Probe and Mach 3 Rabbit HRP-Polymer for ER (10 minutes). Hsp27 (Invitrogen, G3.1), Hsp70 (Invitrogen, MB-H1) and Hsp90 beta (Invitrogen, H9010) were diluted 1:100 in Da Vinci Green Diluent and incubated overnight at 4 °C followed by Mach 3 mouse HRP probe and Mach 3 mouse HRP-polymer incubated for 20 minutes at room temperature.

Chromogen development was performed with Betazoid DAB (Biocare Medical, BDB2004) followed by counterstaining with 20% hematoxylin (Thermo Scientific #7221). Slides were dehydrated in ethanol and xylene and coverslipped with Permount (Fisher Scientific SP15-100). Imaging was performed with a Revolve microscope (Echo Laboratories). Whole image white balancing was performed in Photoshop (Adobe).

### Drug screening

A detailed protocol for the 3D drug screening has been published previously^3,33^. Briefly, the culture medium was fully removed three days after seeding and replaced with 100 µL of Mammocult medium containing the indicated drug treatments using a liquid handler (Eppendorf). After treatment, we transferred the plates to the HSLCI platform for imaging. Staurosporine was tested at concentrations of 0.1, 1, and 10 µM, while neratinib and lapatinib were tested at 0.1, 1, 10, and 50 µM; all drugs were dissolved in DMSO at a final vehicle concentration of 1%. Staurosporine at 1 and 10 µM were used as positive controls, while a 1% DMSO treatment condition was used as a negative control. For HSLCI drug screening experiments, plates were transferred to the HSLCI platform immediately following treatment addition. The content of this article reflects data collected from one HSLCI-based drug screening experiment using organoids derived from each cell line (except for Supplementary Fig. 11) to illustrate the power of the method to gather extensive data from a single well plate. All replicate wells for a given condition were seeded from the same sample preparation and a single cartridge of bioink.

For all drug screening experiments using 2D cultures, the culture medium was removed three days after seeding using the liquid handler and analogously replaced with 100 µL of Mammocult containing the indicated drug treatments. All drug-treated plates were subsequently incubated at 37 °C, 5% CO_2_ for three continuous days. EC_50_ values are reported for all culture conditions for both cell lines in Supplementary Fig. 10 and Supplementary Table 4.

### High-speed live cell interferometry

HSLCI has been described previously^41,42^. The HSLCI platform is a custom-built inverted optical microscope coupled to an off-axis quadriwave lateral shearing interferometry (QWLSI) camera (SID4BIO, Phasics, Inc.)^43^. This wavefront sensing camera incorporates a modified Hartmann mask that splits the incident wave front into four tilted replica wavefronts that interfere with one another. The resulting interferograms are recorded and used to recover phase gradients along two perpendicular directions, allowing for the reconstruction of a phase shift map and subsequent calculation of dry mass of discrete objects within imaging fields of view (FOVs)^43,46^. Measured phase shifts are proportional to mass density^46–48,50,51,122^ and are converted to mass using Eq. (1)^46,47,54^,

1 $$m=\frac{1}{\alpha }\int \phi \lambda {{{{{\rm{d}}}}}}A$$

where *m* is dry biomass, *Φ* is the measured phase shift as a fraction of wavelength, *λ* is illumination wavelength, integration is performed over the area *A* of imaged objects, and *α* is the specific refractive increment, defined as the slope of the relationship between the refractive index and mass concentration of a solution^46,47,50^. Because the specific refractive increments of the biomolecules that constitute most of a cell’s dry mass fall within a narrow range^46,49–51,54,123^, *α* can be assumed to have an average value of 1.8 × 10^−4^ m^3^ kg^−1^ ^46,49–51,54,122–124^.

Illumination is provided by a 660 nm fiber-coupled LED (Thorlabs). The HSLCI platform captures images from a standard-footprint (128 × 85 mm) glass-bottom multiwell plates. Motorized stages (Thorlabs) control the xy-motion of a single glass-bottom plate above the microscope objective and, in combination with a piezo-actuated dynamic focus stabilization system, enable continuous and repeated image collection over many FOVs within each row of wells. The HSLCI platform is installed inside of a standard cell culture incubator to enable long-term imaging of samples in physiology-approximating conditions (37 °C, 5% CO2). All hardware and software components are available commercially.

For all growth kinetics and drug screening studies, organoids were imaged in 96-well glass-bottom plates (Cellvis P96-1.5H-N) using a 40× objective (Nikon, NA 0.75). Plates of bioprinted organoids were prepared as described and wrapped with parafilm to limit evaporation during imaging. Organoids were imaged continuously from 6 hours to 72 hours following the administration of drug treatments. During imaging, the sample plate was translated along each row of wells such that about 25 images per well were collected on each imaging loop, and that imaging FOVs overlapped with areas of wells in which bioprinted matrix and organoids were present. The typical imaging interval was 10 minutes between successive frames at the same FOV.

### Machine learning-based analysis of HSLCI images

Images were acquired using the SID4Bio software (v2.4.2.93, Phasics). After image collection, interferograms captured by the QWLSI camera were converted to phase shift images using the SID4 software development kit for MATLAB (GPU version, v741, Phasics), in a process called phase unwrapping. A custom analysis pipeline was developed in Python and can be run using Nextflow (see the Code Availability section for accession information). For every frame at each FOV, phase shift maps were processed by converting to optical path difference^43^, and then subtracting the fourth-order Zernike polynomial^125^ fit to each frame using least-squares over a cartesian grid to remove refractive aberrations.

The processed images were then segmented into individual cells or organoids using a convolutional neural network (U-Net architecture^72^ with a ResNet-34 encoder^16,73^). The model was initialized with weights derived from a model pretrained on the ImageNet dataset^126^. The training dataset consisted of a randomly selected set of 50 images from the BT-474 dataset, and 50 from the MCF-7 dataset encompassing images taken at all wells, intra-well imaging positions, and timepoints within each experiment. Each 514 × 514 pixel image was overlaid with a binary mask that marks each pixel as either part of an organoid or the background. Using the training dataset, the weights were refined using a cross-entropy loss function over 80 epochs. Phase images and their corresponding masks were organized into one stack per imaging FOV with each layer sorted by time of imaging. TrackMate (v7.6.1)^75,76^ was used to track the organoids over time using a Sparse LAP Tracker with a maximum linking distance of 90 pixels and feature penalties of 4.0 for both the major ellipse axis and organoid area. These parameters were selected based on optimal performance on a set of 12 representative image stacks. Gaps of up to 30 frames were tolerated to maximize track continuity between organoids. Tracks composed of fewer than 10 frames were excluded. Labeled image stacks were exported and mass was extracted from the segmented regions by integrating the phase shift over the area and multiplying by the refractive increment of 1.8 × 10^−4^ m^3^ kg^−1^ ^48,50,51,54,74^.

An XGBoost-based classifier (v1.5.2.1, probability prediction type, log-loss evaluation metric)^77^ was used to predict permissibility of organoid tracks using R package mlr3 (v0.13.2). For the supervised learning model, a subset of the tracks was labelled for permissibility (*n* = 846) from four wells: 1) BT-474 well treated with vehicle, 2) BT-474 well treated with 10 µM staurosporine, 3) MCF-7 well treated with vehicle, and 4) MCF-7 well treated with 10 µM staurosporine. It was manually determined that *n* = 250/846 tracks were acceptable for downstream analysis. A set of time-series based features were extracted from the mass reconstruction data to use in our classifier model. The features were number of missing frames, initial size, interquartile range (IQR), IQR of the first 12 points, and IQR of the last 12 points (Supplementary Fig. 6). Initial size was calculated based on the median mass of the first two timepoints. Area under the curve (AUC) measurements were calculated using R package (bayestestR v0.11.5) (Supplementary Fig. 12). Pair-wise correlation plots comparing the void and valid track features were generated using mlr3viz R package (v0.5.7) (Supplementary Fig. 6). Probability was used as the learner prediction type and log-loss (log_10_) as the evaluation metric. All default hyperparameters were used (Supplementary Table 1). To validate the model, 3-fold cross-validation with resampling was performed. The trained model had excellent accuracy and specificity with an AUC of 0.966 ± 0.020 and a cross-validation score of 93.1 ± 1.3% (Supplementary Table 2). The model was applied to the whole dataset and predicted *n* = 8,590/29,137 tracks to be permissible for downstream analysis.

### ATP release assay

Manually seeded organoids, bioprinted organoids, and 2D-cultured cell lines were prepared in accordance with the protocols described above and published^3,16,33^. For drug screenings, plates were retrieved from the HSLCI incubator and processed as briefly described. After a PBS wash, 50 µL of 5 mg/mL Dispase (Life Technologies 17105-041) solution was added to each well and incubated for 25 minutes. After shaking for 5 minutes on an orbital shaker (Corning 6780-4) at 800 RPM, we added 30 µL of CellTiter-Glo® Luminescent Cell Viability Reagent (Promega G968B) to each well and followed the manufacturer’s instructions. For 2D drug screening experiments, 30 µL per well of CellTiter-Glo® 3D Luminescent Cell Viability Reagent (Promega G968B) was added directly to the drug-treated media at the conclusion of the experiment. Luminescence was measured using a SpectraMax iD3 (Molecular Devices) plate reader (parameters: read all wavelengths, signal integration of 500 ms). The viability of each well was calculated by normalizing the luminescent signal to the average signal from the manually seeded control wells. An unpaired t-test with Welch’s correction was performed in Prism 9 (GraphPad). *p*-values less than 0.05 were deemed significant. EC_50_ values for each drug-organoid model combination were computed by interpolating the drug concentrations that would yield 50% relative viability in treated organoids. For each drug, results from all tested concentration conditions were used to compute EC_50_ values from ATP-catalyzed luminescence data, including from 50 µM neratinib-treated wells that were excluded from HSLCI data analysis.

To assess the viability of bioprinted organoids (as shown in Fig. 1d), the bioink and bioprinter were prepared as previously described. Then, 100 µL of bioink was extruded into an Eppendorf tube for each print pressure (10, 15, 20, and 25 kPa). Using a pipette, four 10 µL rings were seeded in a 96-well plate using the extruded bioinks and non-extruded bioink from the same batch. Cells were then released from Matrigel, CellTiter-Glo® 3D Luminescent Cell Viability Reagent (Promega G968B) was added to each well, and ATP-catalyzed luminescence values were obtained as described above. The viability of each well was calculated by normalizing its luminescence signal to the average luminescence signal from the manually seeded control wells. Statistical analyses were performed in Prism 9 (GraphPad). *p*-values less than 0.05 were deemed significant.

### Statistics and reproducibility

At the end of each set of experiments, cell identity was confirmed by STR profiling (Laragen). Three independent bioprinting experiments per cell line were used to evaluate organoid properties using IHC, with similar results. We pooled *n* = 4 maxi rings for each condition with the exception of BT-474 24h from experiment #2, with n = 3 and proceeded to the library preparation and sequencing for RNAseq experiments. This was repeated on two independent sets of samples by two different operators, and data was aggregated for analysis. HSLCI data presented in Figs. 3–5 shows an individual experiment for each cell line to demonstrate the quantity of information that can be gathered from a single run. Each HSLCI experiment was repeated a minimum of two times, with similar results as visible in Supplementary Fig. 11 which shows a comparison of two independent BT-474 organoid HSLCI datasets. Statistical tests are described within figure captions, where applicable, with exact *p*-values listed either in the figure legends or within supplementary tables. A significance threshold of *p* < 0.05 was used for all statistical tests. No statistical method was used to predetermine the sample size. We excluded neratinib 50 µM HSLCI data points from the analysis because significant drug precipitates deteriorate image quality to the point the data measured is not quantitative and thus unusable for image analysis purposes.

### Reporting summary

Further information on research design is available in the Nature Portfolio Reporting Summary linked to this article.

## Supplementary information

Supplementary Information

Reporting Summary

Description of Additional Supplementary Files

Supplementary Video 1

Supplementary Video 2

## Source data

Source Data

## Data Availability

HSLCI imaging data and training datasets as presented in Figs. 3–5 are deposited in the BioImage Archive on Biostudies under accession code S-BIAD616. Sequencing data is deposited and available on GEO under accession number GSE218693. Source data for all figure components are provided with this paper. Source data are provided with this paper.

## Code availability

Code for the data analysis pipeline is publicly available on GitHub at https://github.com/uclahs-soragnilab/hslci_pipeline or Zenodo^127^ (10.5281/zenodo.7787339). A ready-to-use, publicly available Docker image is hosted on Dockerhub at: https://hub.docker.com/r/soragnilab/unetsegmentation.
