# Supplementary material for: Drug screening at single-organoid resolution via bioprinting and interferometry
Source: Nat Commun. 2023 Jun 6;14:3168. doi: 10.1038/s41467-023-38832-8 (PMC10244450; doi:10.1038/s41467-023-38832-8)
Supplement: Supplementary file 1 — Supplementary Information [file 41467_2023_38832_MOESM1_ESM.pdf]

Supplementary Information

Drug screening at single-organoid resolution via bioprinting and interferometry

Peyton J. Tebon*, Bowen Wang*, Alexander L. Markowitz*, Ardalan Davarifar, Brandon L. Tsai, Patrycja Krawczuk, Alfredo Enrique Gonzalez, Sara Sartini, Graeme F. Murray, Huyen Thi Lam Nguyen, Nasrin Tavanaie, Thang L. Nguyen, Paul C. Boutros, Michael A. Teitell#, Alice Soragni#

Supplementary Figures

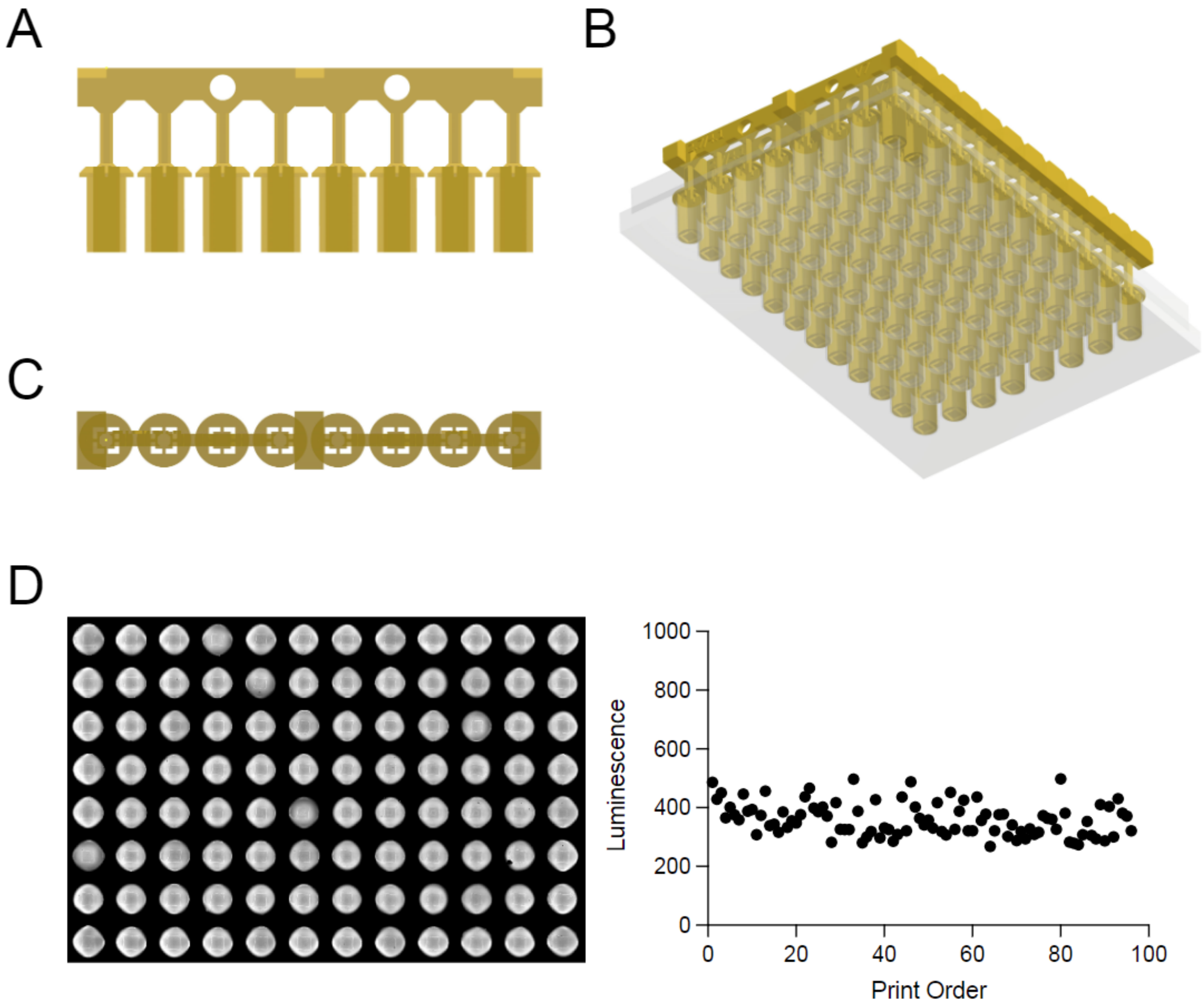

Figure S1: Schematics of well mask and representative bioprint and assessment of bioprinting consistency. (A) Side view of plasma masks. (B) Bottom view of plasma masks. (C) Plasma masks inserted into 96-well plate as viewed from the bottom. (D) Stitched, contrast-enhanced brightfield images of a a 96-well glass bottom plate with bioprinted cell-laden Matrigel bioink. An ATP-release assay was performed immediately after printing, with data plotted on the right. The coefficient of variation (CV%) for the plate shown is 15.6%. The average CV% for bioprinted plates (20.3±6.9) and manually seeded plates (13.8±7.0) used in this study are not statistically different (p=0.34, Mann-Whitney).

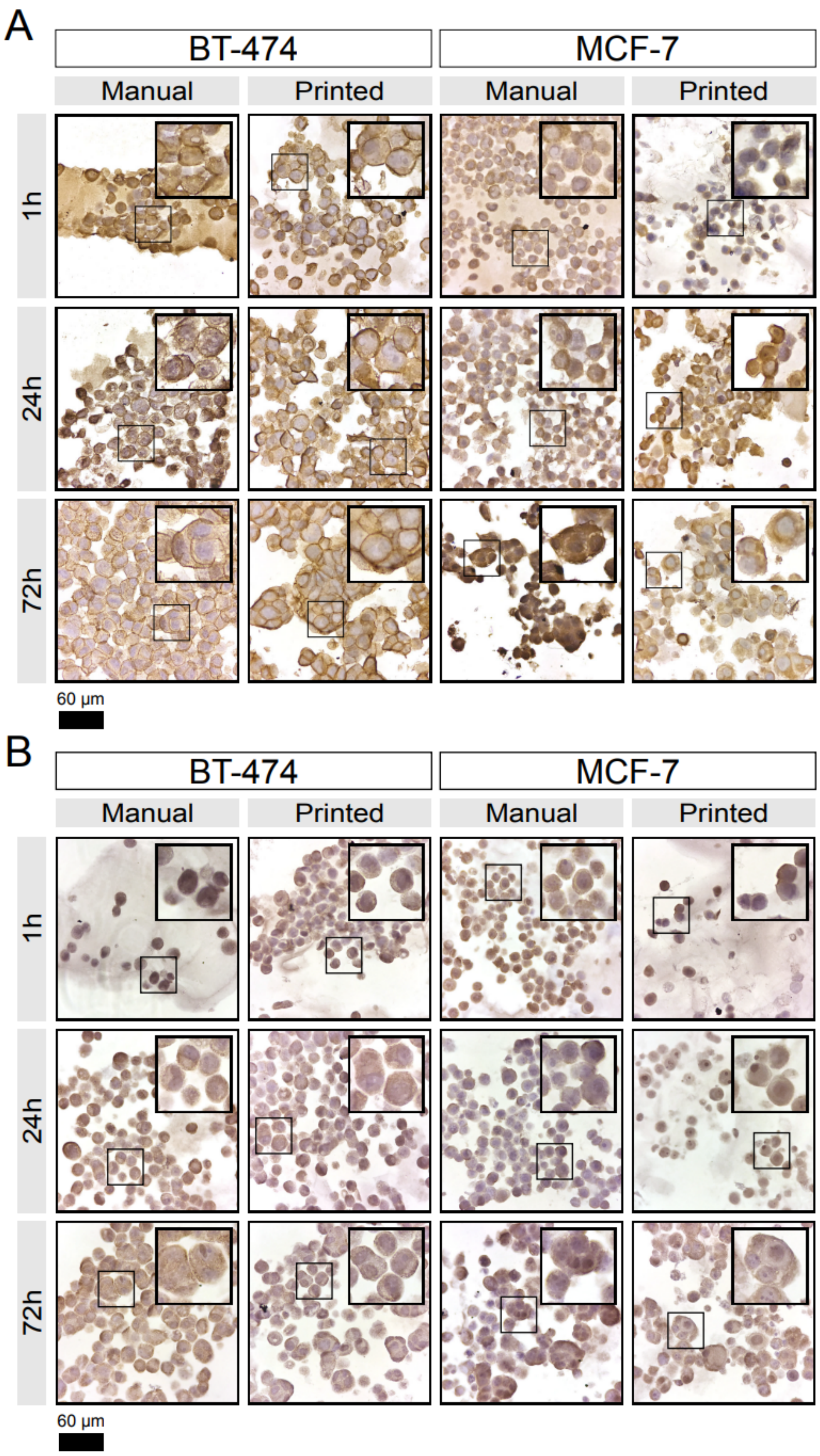

Figure S2: HER2 and ER expression in BT-474 and MCF-7 lines is not altered by bioprinting. (A) Immunohistochemistry staining of 3D cultures for HER2. BT-474 cells have amplified HER2 expression while MCF-7 cells express lower levels of HER2 and lack HER2 amplification. (B) Immunohistochemistry staining of 3D cultures for ER. Both BT-474 and MCF-7 cell lines are ER-positive.

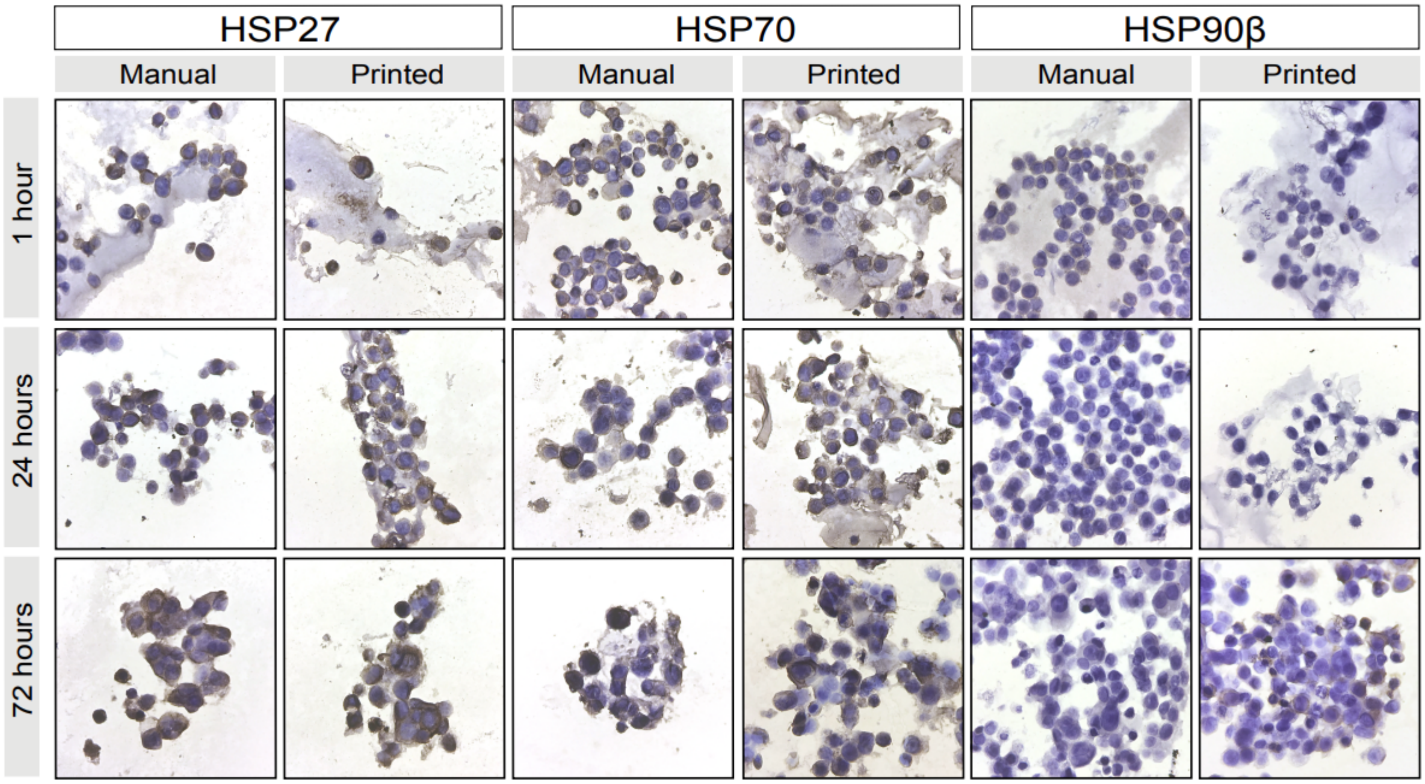

Figure S3: Chaperone expression is not altered by bioprinting. Comparison of HSP27, HSP70 and HSP90 staining in BT474 cells 1, 24 and 72 hours after manually seeding or bioprinting. We found no detectable difference in protein levels.

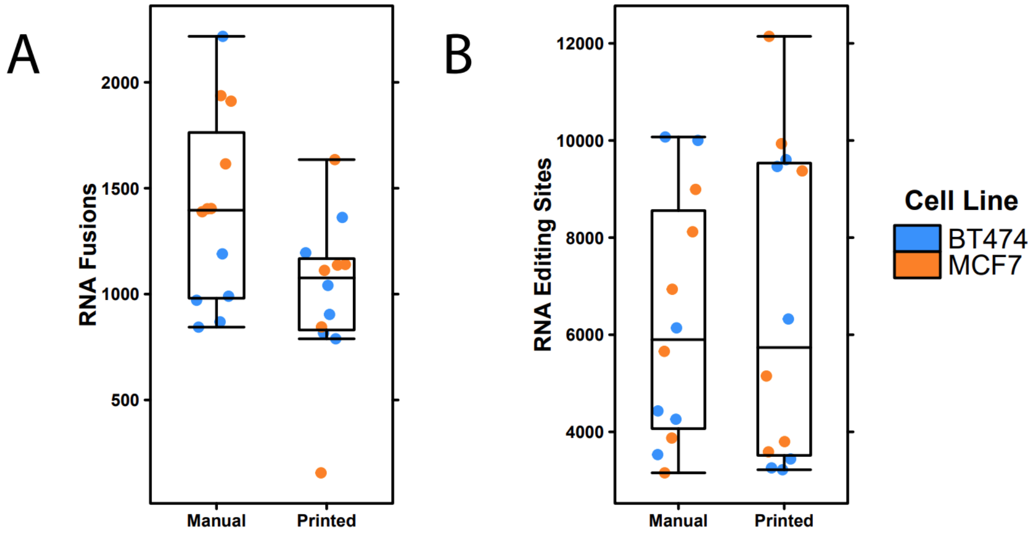

Figure S4: Bioprinting did not significantly alter the number of RNA Fusions or Editing Sites. (A) Number of RNA fusions detected by FusionCatcher. The number of RNA fusions did not significantly differ between manually seeded and bioprinted organoids ($p = 0.0519$, Mann-Whitney U-test). (B) Number of adenosine-to-inosine (A-to-I) RNA editing sites detected by REDItools2. The number of RNA editing sites did not significantly differ between manually seeded and bioprinted organoids ($p = 0.977$).

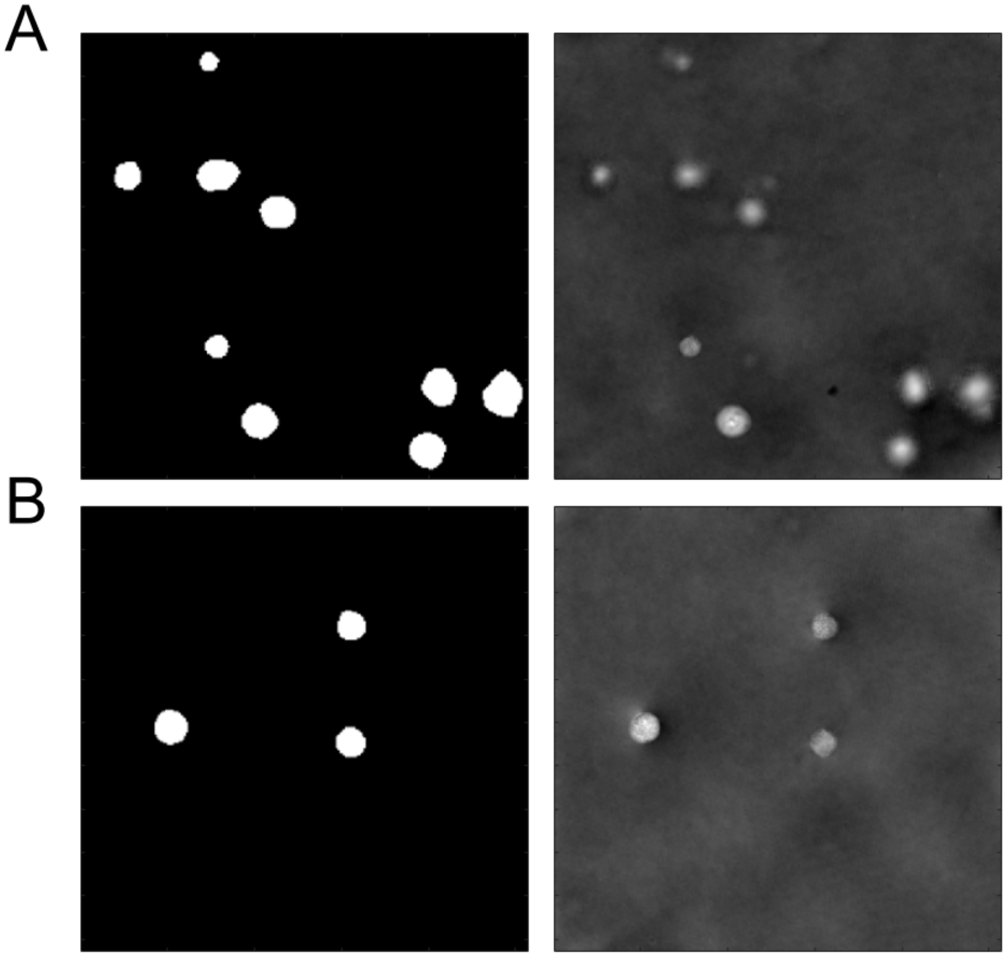

Figure S5: Image segmentation using a U-Net convolutional neural network. Representative masks (left) predicted by the U-Net-based segmentation algorithm for the background-corrected phase images (right).

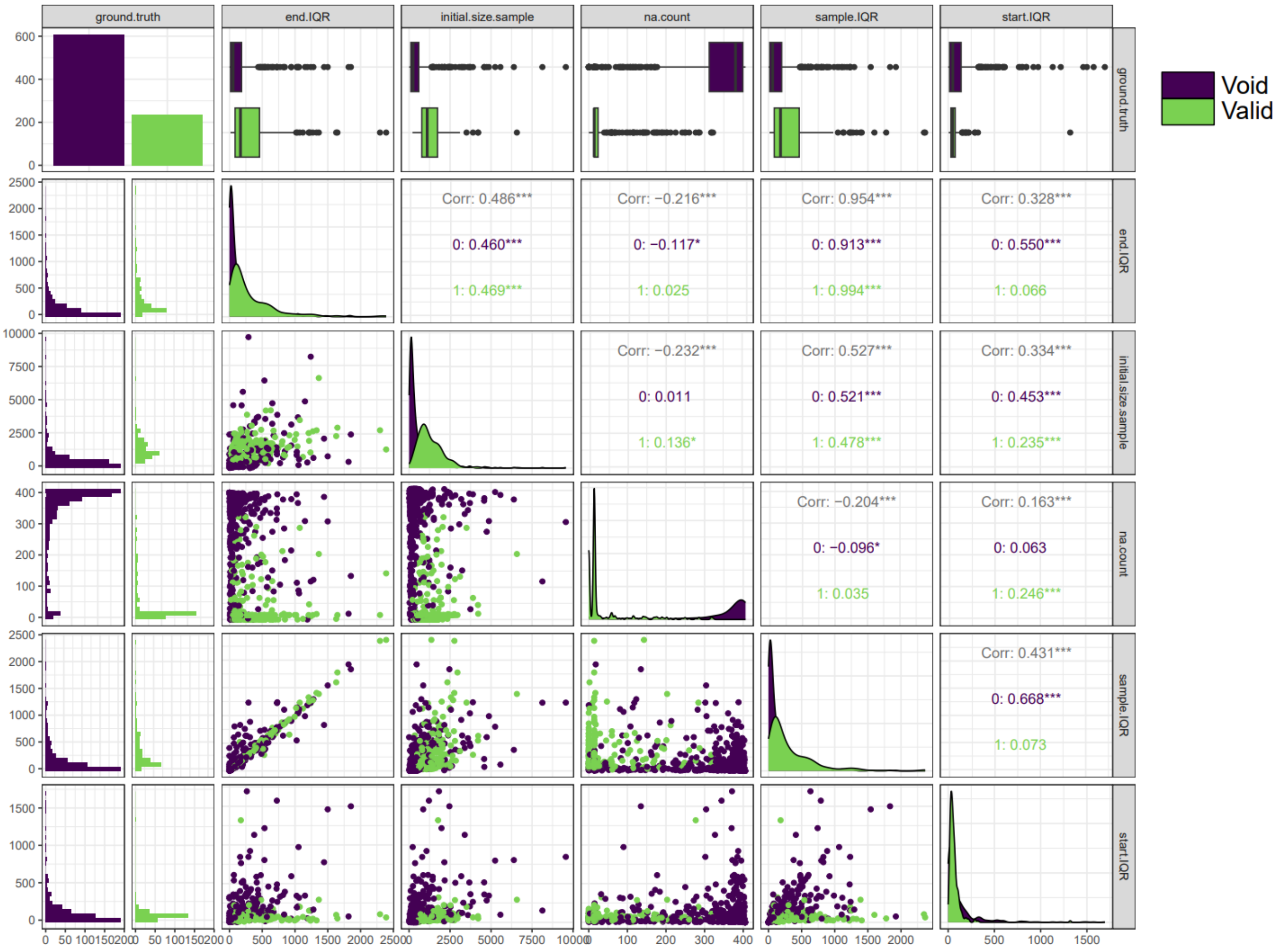

Figure S6: Pair-wise correlation matrix of classifier data. Extracted time-series analytical features of tumor growth patterns over recorded time. The number of missing frames (na.count), initial mass (initial.size.sample), interquartile range (sample.IQR), and interquartile range of the first and last 12 data points (start.IQR and end.IQR) were measured for each tracked tumor organoid. We labelled (n = 250 out of 846) tracked organoids as valid for downstream analysis. Pair-wise correlations are shown of the valid (label = 1, green) and void (label = 0, purple) tracked organoids. Void tracks showed an increased number of missing frames, smaller overall IQR, and smaller initial size. Correlation of the classification data are shown within the paired subplots. Overall and end IQR were strongly correlated (R^2 = 0.954). An XGBoost classifier was used to train a model to classify organoids as valid or void. We validated the model via cross-validation with 3-fold resampling of the sample population. The cross-validation score of the classifier was 93.1% with 96.6% AUC (**Table S2**).

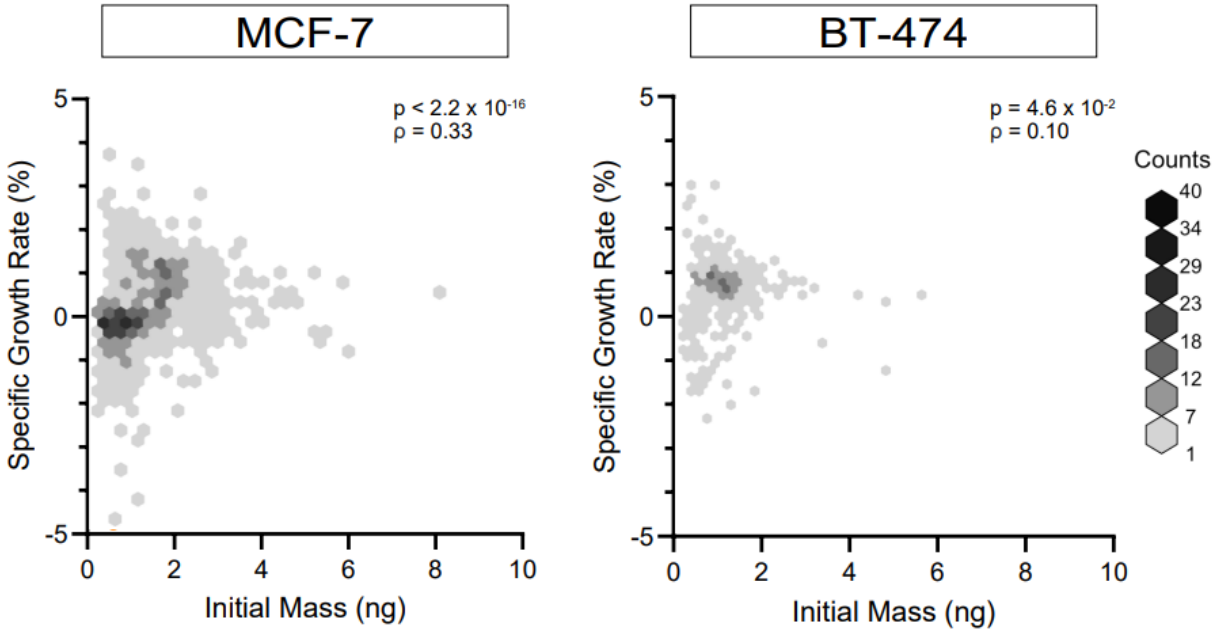

Figure S7: Specific growth rate correlates to initial organoid mass. Specific growth rate (growth in mass as a percentage of total mass) versus initial organoid mass was plotted for all organoids tracked. For both MCF-7 and BT-474 cell line-derived organoids, we observed a positive relationship between initial organoid mass and specific growth rate. This association is stronger for MCF-7 cells (Spearman's $\rho = 0.33$, $p < 2.2 \times 10^{-16}$) compared to BT-474 ($\rho = 0.10$, $p = 4.6 \times 10^{-2}$).

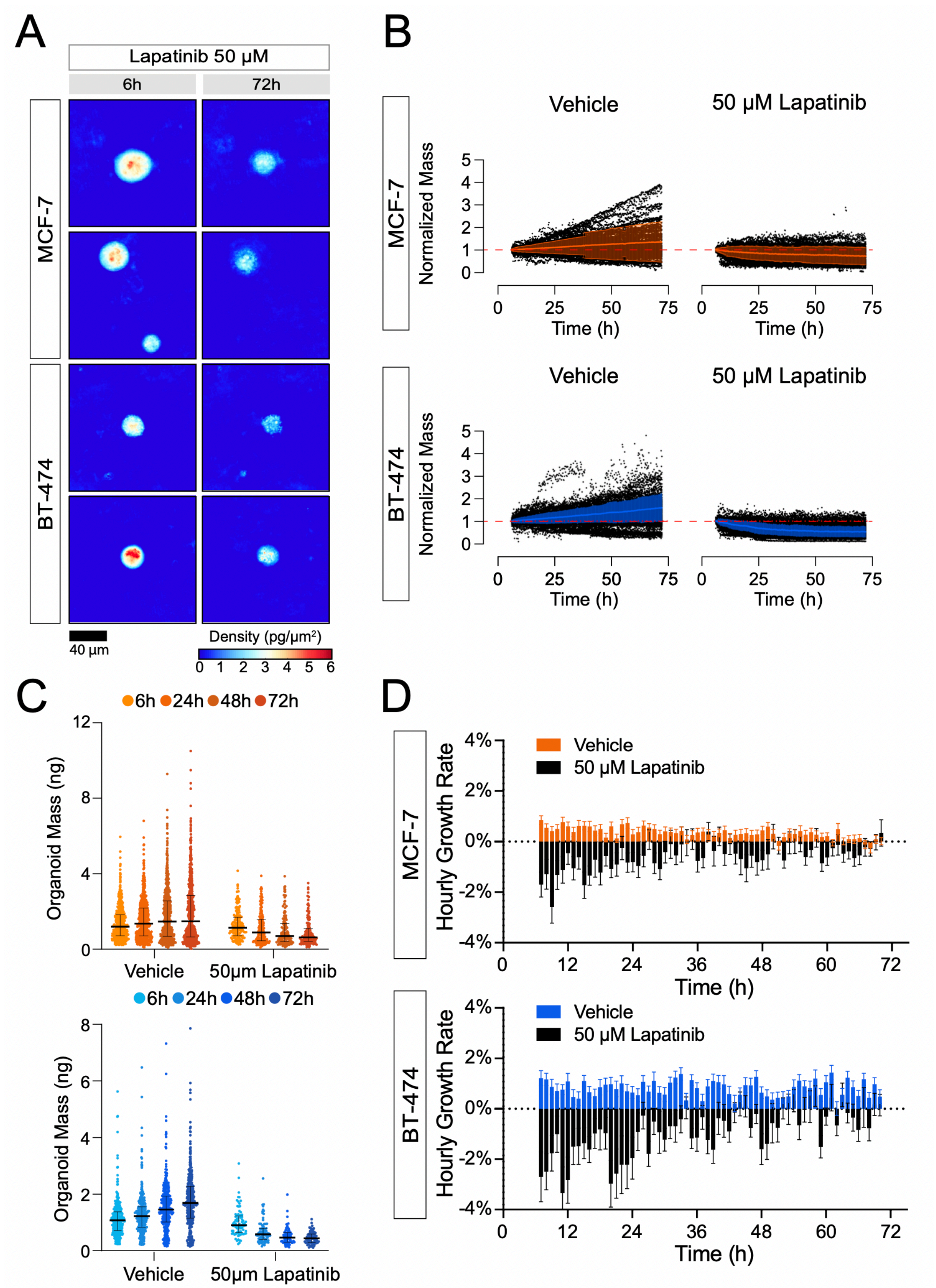

Figure S8: Response of bioprinted organoids to 50 μM lapatinib. (A) Representative HSLCI-acquired phase images of organoids treated with 50 μM lapatinib. (B) The mean normalized mass ± standard deviation is shown in orange (MCF-7) and blue (BT-474). 100 organoid tracks for are shown for each plot. (C) Mass distribution of tracked MCF-7 and BT-474 by treatment. Each column represents the mass distribution at 6-, 24-, 28-, and 72-hours post-treatment (left to right). Black horizontal bars represent the median with error bars representing the interquartile range of the distribution. (D) Hourly growth rate comparisons (percent mass change) between organoids treated with 50 μM lapatinib and vehicle.

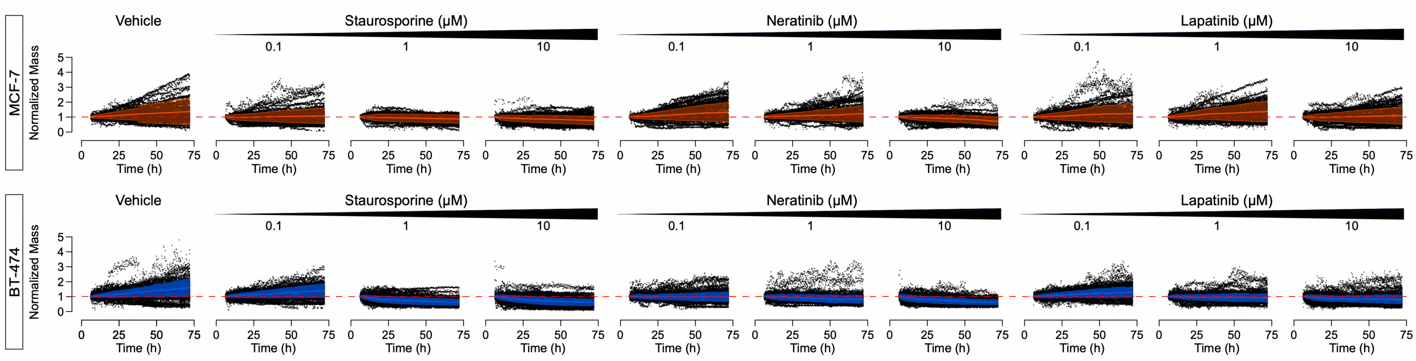

Figure S9: Representative normalized mass tracks by treatment condition. The mean normalized mass ± standard deviation is also shown in orange (MCF-7) and blue (BT-474). 100 organoid tracks for each treatment condition are shown for each plot.

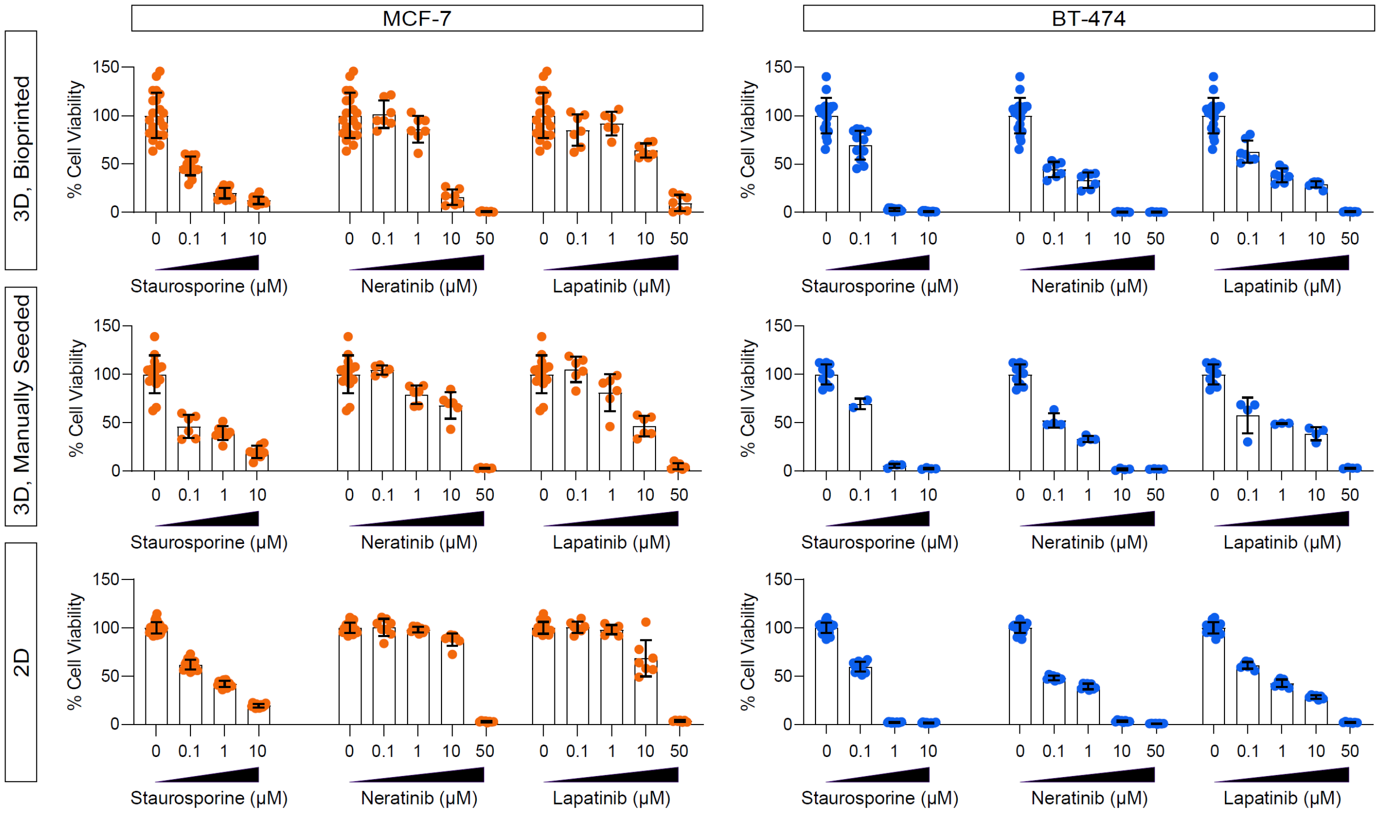

Figure S10: ATP assay readout in multiple culture conditions. ATP-release assay quantifies cell viability for staurosporine, neratinib, and lapatinib-treated MCF-7 and BT-474 breast cancer models in 3D culture following bioprinting, 3D culture following manual seeding, or 2D monolayer cell culture. Data shown were merged from a minimum of n=2 independent experiments. Bars represent mean values, and error bars represent standard deviation (SD).

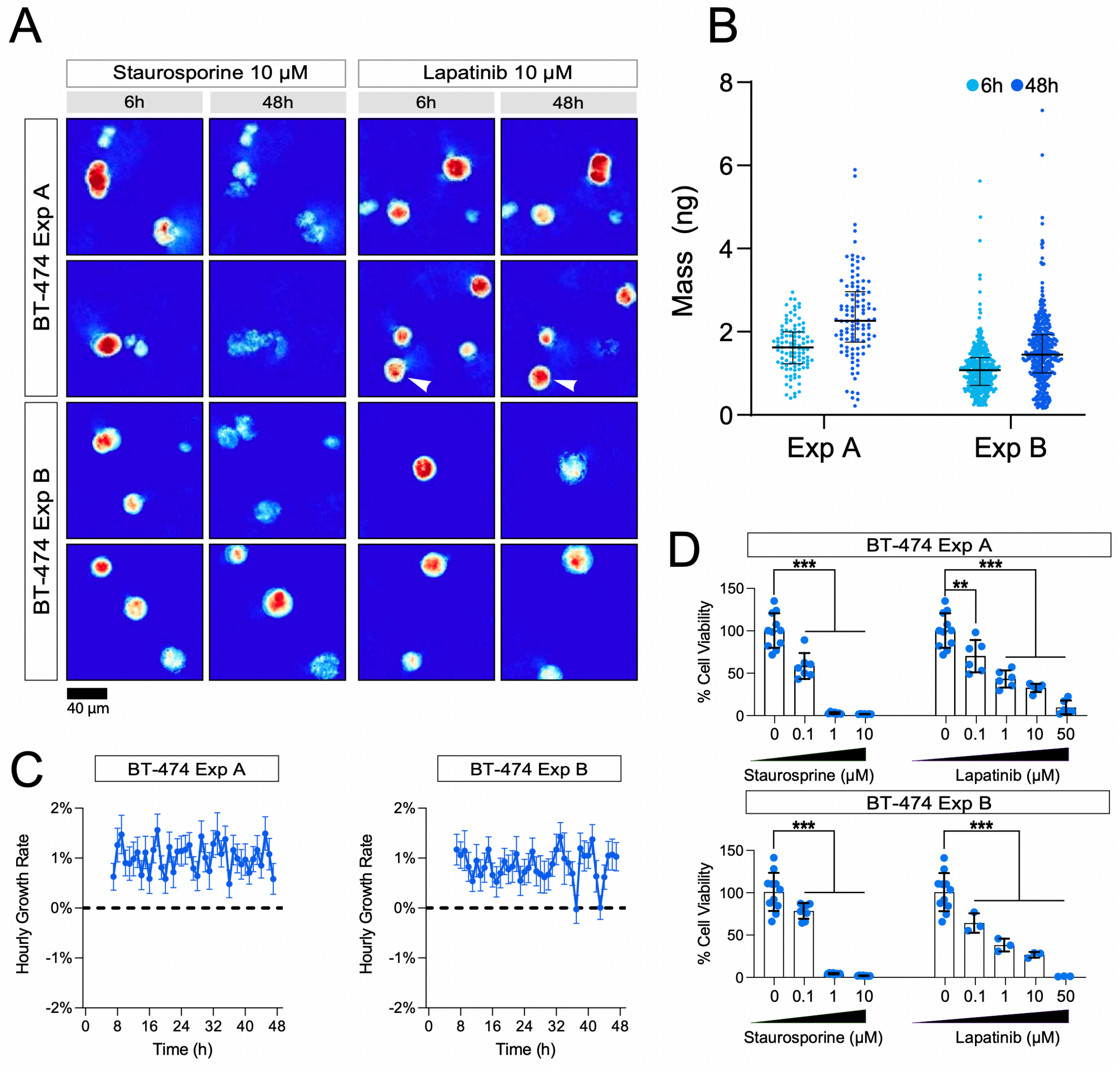

Figure S11: Comparison of BT-474 organoid datasets. Experiment A and B were independently performed. followed the same protocols with three exceptions. Experiment A had a shorter total duration (48h of HSLCI imaging) and was analyzed with a legacy pipeline (see Tebon et al v1, biorXiv 2021). (A) Representative images of BT-474 organoids treated with 10 μM staurosporine and 10 μM lapatinib. The white arrow indicates a treatment-resistant organoid. (B) Mass distribution of tracked BT-474 organoids by treatment. The left column (pale blue) represents the mass distribution 6 hours post-treatment, while the right column (dark blue) represents the mass distribution 48 hours after treatment. Black horizontal bars represent the median with error bars showing the interquartile range of the distribution. (C) Hourly growth rate comparisons (percent mass change per hour) between 50 μM lapatinib and vehicle-treated cells. (D) An ATP-release assay was performed on the same plates imaged by HSLCI at the end of imaging. Statistical significance was assessed using an unpaired t-test with Welch's correction. $p<0.05$ is denoted by *, $p<0.01$ is denoted by **, and $p<0.001$ is denoted by ***.

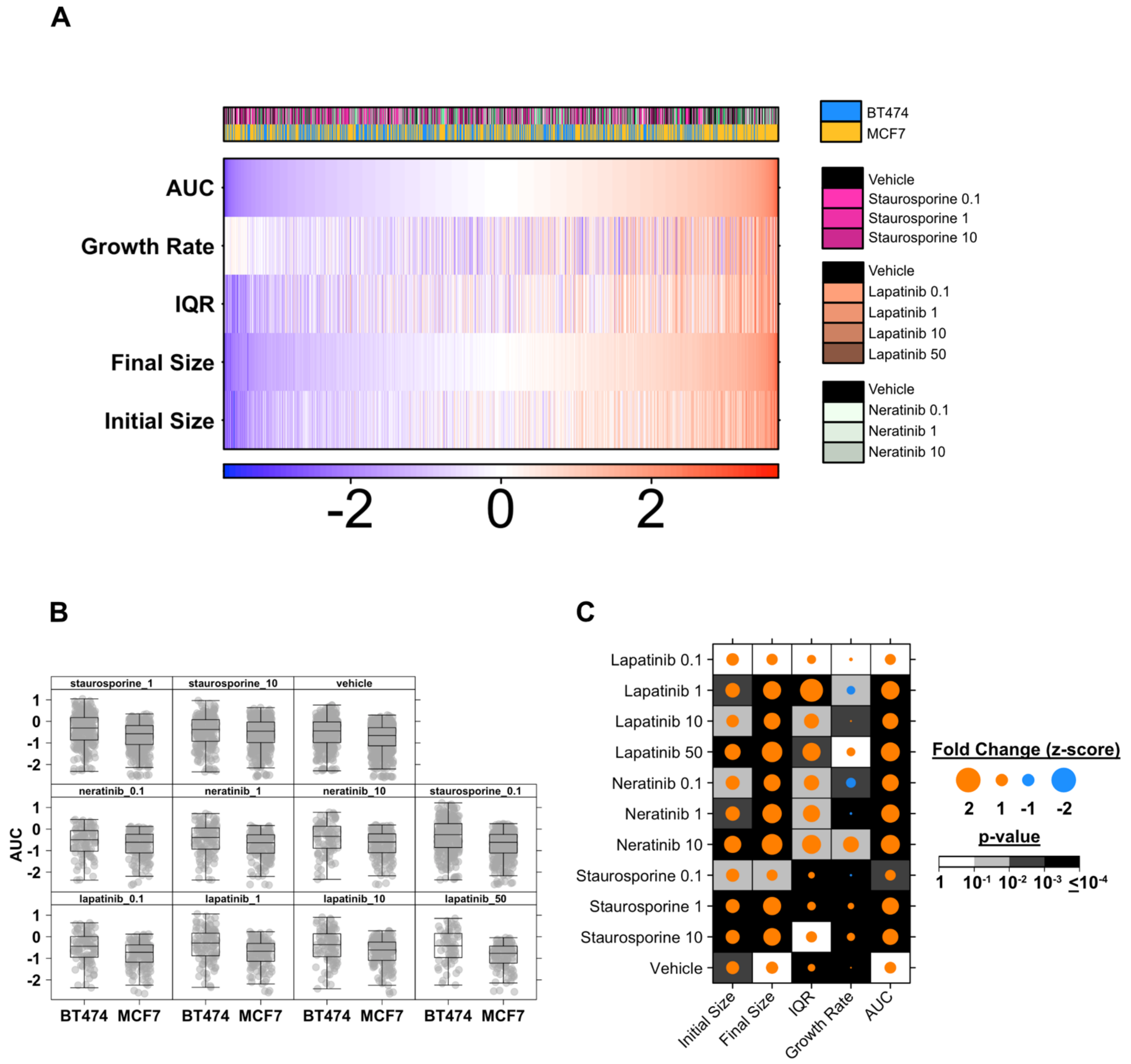

Figure S12: Growth patterns of MCF-7- and BT-474 upon pharmacological treatments. We assessed the 3D growth patterns of MCF-7 and BT-474 breast cancer cell lines. Both cell lines were treated with various concentrations of lapatinib, neratinib and staurosporine ranging from 0.1 to 10 μM. (A) Growth patterns of tumor organoids are arranged by area under the curve (AUC) metric measured by the integration of a fitted time-series step function. Z-transformed measurements of organoid AUC, linear growth pattern (R^2 of a linear fit), initial size, final size and interquartile range (IQR) varied among sample population. (B) Overall growth patterns measured as AUC differed among each treatment condition. (C) Fold change of growth patterns features were found to be significantly different among MCF-7 and BT-474 in all conditions.

Supplementary Results

Mass reconstruction data extracted for classifier

Mass reconstruction data of n = 8,590 tracked BT-474 and MCF-7 breast cancer cell line-derived organoids were assessed for change in mass over 72 hours following treatment. BT-474 and MCF-7 lines were treated with a series of concentrations of 0.1 to 50 μM of vehicle (n = 1,593), lapatinib (n = 1,650), neratinib (n = 1,626) and staurosporine (n = 2,920). We assessed the cell cluster growth patterns using z-transformed measurements of area under the curve (AUC), linear growth rate, interquartile range, initial and final mass (**Figure S12A**). Sampled BT-474 and MCF-7 under various pharmacological treatments display a diverse set of growth patterns, arranged by AUC metric. Cell line and treatment type were dispersed across the sample population, thus supporting our choice to use a single set of classifier training data for both cell lines and all treatment conditions. We tested whether cell clusters derived from BT-474 cells displayed differences in their growth patterns compared to clusters derived from MCF-7 cells (**Figure S12B**). We found significant differences in growth patterns of BT-474 and MCF-7 across treatments (**Figure S12C**). In most conditions (9 out of 11 conditions), the z-transformed initial size, final size and linear growth rate of MCF-7 clusters were larger than BT-474 in all three pharmacological treatment conditions (**Figure S12B-C**) ($p < 0.0001$, Mann-Whitney U-test). In two conditions, the linear growth rate of BT-474 in the presence of 1 μM lapatinib (fold change = -0.70, $p = 2.76 \times 10^{-2}$) and 0.1 μM neratinib (fold change = -0.82, $p = 3.82 \times 10^{-3}$) were found to be greater than MCF-7. We did not find strong differences in growth patterns among BT-474 and MCF-7 treated with 0.1 μM lapatinib.

Supplementary Tables

Parameter Name	Setting
alpha	0
approxcontrib	FALSE
base_score	0.5
booster	gbtree
colsample_bylevel	1
colsample_bynode	1
colsample_bytree	1
disable_default_eval_metric	FALSE
eta	0.3
feature_selector	cyclic
gamma	0
grow_policy	depthwise
lambda	1
lambda_bias	0
max_bin	256
max_delta_step	0
max_depth	6
max_leaves	0
min_child_weight	1
missing	NA
monotone_constraints	0
normalize_type	tree
nthread	1
num_parallel_tree	1

Parameter Name	Setting
objective	binary:logistic
one_drop	FALSE
outputmargin	FALSE
predcontrib	FALSE
predictor	cpu_predictor
predinteraction	FALSE
predleaf	FALSE
print_every_n	1
process_type	default
rate_drop	0
refresh_leaf	TRUE
reshape	FALSE
seed_per_iteration	FALSE
sample_type	uniform
sampling_method	uniform
scale_pos_weight	1
skip_drop	0
strict_shape	FALSE
subsample	1
top_k	0
training	FALSE
tree_method	auto
tweedie_variance_power	1.5
verbose	1

Table S1: Hyperparameters used for XGBoost Classifier. The default hyperparameters were used for all settings.

Resampling	Accuracy	AUC
1	0.926	0.967
2	0.922	0.945
3	0.947	0.984
Average ± SD	0.931 ± 0.013	0.966 ± 0.020

Table S2: 3-fold resampling cross-validation scores of the XGBoost classifier. We report model accuracy as the average accuracy and AUC of the three resampled test sets.

A

		6 hours	24 hours		48 hours		72 hours	
MCF-7		Mean mass ± SD	Mean mass ± SD	p-value	Mean mass ± SD	p-value	Mean mass ± SD	p-value
Vehicle	1% DMSO	1.36 ± 0.84	1.56 ± 1.05	-	1.77 ± 1.33	-	1.95 ± 1.61	-
Staurosporine	0.1 µM	1.30 ± 0.84	1.27 ± 0.89	2.11×10^{-5}	1.39 ± 1.07	1.31×10^{-5}	1.44 ± 1.21	2.49×10^{-6}
	1 µM	1.28 ± 0.84	1.18 ± 0.77	1.93×10^{-9}	1.10 ± 0.73	2.24×10^{-17}	1.03 ± 0.70	2.80×10^{-21}
	10 µM	1.30 ± 0.78	1.11 ± 0.69	1.49×10^{-13}	1.01 ± 0.65	6.73×10^{-24}	0.93 ± 0.64	3.24×10^{-30}
Neratinib	0.1 µM	1.47 ± 1.03	1.62 ± 1.18	1	1.86 ± 1.51	1	2.03 ± 1.74	1
	1 µM	1.45 ± 0.96	1.57 ± 1.09	1	1.68 ± 1.22	1	1.79 ± 1.40	1
	10 µM	1.39 ± 1.07	1.30 ± 0.98	0.0036	1.16 ± 0.92	2.90×10^{-9}	0.92 ± 0.77	6.82×10^{-19}
Lapatinib	0.1 µM	1.38 ± 1.03	1.49 ± 1.19	0.9072	1.73 ± 1.47	1	1.79 ± 1.56	1
	1 µM	1.40 ± 0.83	1.61 ± 1.11	1	1.84 ± 1.38	1	2.02 ± 1.69	1
	10 µM	1.29 ± 0.83	1.30 ± 0.98	0.0011	1.42 ± 1.11	0.0013	1.50 ± 1.29	0.0006
Kruskal-Wallis p-value		0.3169	2.21×10^{-22}		4.57×10^{-45}		5.13×10^{-60}	

B

		6 hours		24 hours		48 hours		72 hours	
BT-474		Mean mass ± SD	p-value	Mean mass ± SD	p-value	Mean mass ± SD	p-value	Mean mass ± SD	p-value
Vehicle	1% DMSO	1.12 ± 0.61	-	1.27 ± 0.69	-	1.52 ± 0.84	-	1.77 ± 1.00	-
Staurosporine	0.1 µM	1.10 ± 0.57	1	1.22 ± 0.67	1	1.39 ± 0.82	0.0756	1.56 ± 0.96	0.0093
	1 µM	1.02 ± 0.53	0.0901	0.76 ± 0.39	2.27×10^{-32}	0.63 ± 0.34	1.28×10^{-57}	0.57 ± 0.32	3.03×10^{-62}
	10 µM	1.02 ± 0.51	0.1342	0.79 ± 0.44	1.59×10^{-28}	0.63 ± 0.36	4.55×10^{-56}	0.56 ± 0.32	2.52×10^{-62}
Neratinib	0.1 µM	1.04 ± 0.46	1	0.97 ± 0.44	4.86×10^{-6}	0.97 ± 0.45	5.09×10^{-14}	0.98 ± 0.46	6.93×10^{-18}
	1 µM	1.08 ± 0.54	1	0.98 ± 0.49	4.34×10^{-7}	0.86 ± 0.40	1.19×10^{-20}	0.81 ± 0.36	1.48×10^{-27}
	10 µM	0.92 ± 0.42	0.0043	0.70 ± 0.31	3.73×10^{-21}	0.59 ± 0.28	1.99×10^{-31}	0.53 ± 0.21	4.71×10^{-34}
Lapatinib	0.1 µM	1.05 ± 0.52	1	1.13 ± 0.57	0.2512	1.23 ± 0.61	0.0025	1.31 ± 0.67	1.83×10^{-5}
	1 µM	1.08 ± 0.54	1	1.00 ± 0.49	3.37×10^{-5}	0.96 ± 0.45	1.80×10^{-14}	0.93 ± 0.43	1.70×10^{-21}
	10 µM	1.09 ± 0.47	1	0.91 ± 0.45	7.72×10^{-10}	0.86 ± 0.40	3.31×10^{-20}	0.83 ± 0.41	2.13×10^{-24}
Kruskal-Wallis p-value		0.0007		4.25×10^{-70}		1.38×10^{-141}		7.68×10^{-168}	

Table S3: Mass distributions for MCF-7 and BT-474 cancer cells grown in 3D. (A) Comparisons of mean mass of MCF-7 cells and cell clusters calculated via HSLCI. (B) Comparisons of mean mass of BT-474 cells and cell clusters calculated via HSLCI. For both (A) and (B), statistical significance was assessed using Kruskal-Wallis tests. For samples with Kruskal Wallis test p-values lower than 0.05, we performed Mann-Whitney U-tests against the vehicle control at the respective time points. Data is presented in Figure 4B.

		MCF-7				BT-474			
		3D, Bioprinted	3D, Manually Seeded	2D		3D, Bioprinted	3D, Manually Seeded	2D	
Drug	C_{max}	EC_{50} ± SD	EC_{50} ± SD	EC_{50} ± SD	Kruskal-Wallis p-value	EC_{50} ± SD	EC_{50} ± SD	EC_{50} ± SD	Kruskal-Wallis p-value
Staurosporine	-	0.08 ± 0.03	0.28 ± 0.30	0.40 ± 0.01	0.36	0.14 ± 0.05	0.08 ± 0.11	0.12 ± 0.01	0.89
Neratinib	0.15 μM	3.28 ± 0.26	16.54 ± 0.82	29.61 ± 0.62	0.07	0.08 ± 0.05	0.15 ± 0.07	0.12 ± 0.01	0.80
Lapatinib	4.2 μM	12.60 ± 1.27	9.57 ± 4.43	16.67 ± 7.58	0.80	0.48 ± 0.03	1.07 ± 0.96	0.59 ± 0.26	1

Table S4: EC_{50} values of MCF-7 and BT-474 breast cancer models in multiple culture conditions. Comparisons of staurosporine, neratinib, and lapatinib treatment efficacy on MCF-7 and BT-474 breast cancer models in 3D culture following automatic seeding using bioprinting, 3D culture following manual seeding, or 2D monolayer cell culture, as measured by ATP-release assay. Data were obtained from two to three independent experiments. All concentration values are shown in μM. EC_{50} values were computed using data points from all tested concentration conditions, including 50 μM neratinib that was excluded from HSLCI analyses due to drug precipitation that resulted in unquantifiable phase images. Statistical significance was assessed using Kruskal-Wallis tests. For all model-drug combinations, no significant differences were observed in average EC_{50} values among different culturing methods. The peak serum concentrations (C_{max}) of neratinib and lapatinib as reported in Keyvanjah et al, 2017 and Liston et al, 2017 respectively are included for reference.

A

MCF-7		Time (h)	n	Organoid Behavior (%) Gained Mass	Maintained Mass	Lost Mass
Vehicle	1% DMSO	12	800	23.2	67.4	9.4
		24	794	44.8	36.0	19.1
		48	764	48.6	24.1	27.4
		72	715	50.8	16.6	32.6
Staurosporine	0.1 µM	12	481	8.7	72.3	18.9
		24	481	21.8	46.8	31.4
		48	458	31.7	27.3	41.0
		72	436	32.8	20.4	46.8
	1 µM	12	508	10.2	74.6	15.2
		24	505	11.7	56.6	31.7
		48	490	13.7	36.9	49.4
		72	460	10.0	31.3	58.7
	10 µM	12	539	6.9	64.7	28.4
		24	538	8.4	40.3	51.3
		48	523	9.0	22.9	68.1
		72	494	8.3	16.2	75.5
Neratinib	0.1 µM	12	205	19.5	67.3	13.2
		24	206	43.7	38.8	17.5
		48	196	49.5	27.0	23.5
		72	184	49.5	21.7	28.8
	1 µM	12	222	18.0	71.6	10.4
		24	221	37.6	45.2	17.2
		48	214	44.4	30.8	24.8
		72	203	46.3	24.6	29.1
	10 µM	12	223	14.3	68.6	17.0
		24	219	12.8	51.1	36.1
		48	213	8.0	34.3	57.7
		72	199	4.5	18.1	77.4
Lapatinib	0.1 µM	12	218	21.1	66.5	12.4
		24	214	35.5	43.5	21.0
		48	203	42.9	24.6	32.5
		72	187	43.3	22.5	34.2
	1 µM	12	202	17.8	69.8	12.4
		24	202	47.5	33.2	19.3
		48	190	55.3	21.1	23.7
		72	177	53.7	21.5	24.9
	10 µM	12	251	15.5	62.5	21.9
		24	252	32.5	31.0	36.5
		48	238	36.1	18.9	45.0
		72	220	37.7	10.0	52.3

B

BT-474		Time (h)	n	Organoid Behavior (%) Gained Mass	Maintained Mass	Lost Mass
Vehicle	1% DMSO	12	419	30.1	59.9	10.0
		24	415	62.4	26.7	10.8
		48	409	80.9	10.8	8.3
		72	387	80.9	7.2	11.9
Staurosporine	0.1 µM	12	335	31.0	55.2	13.7
		24	334	49.7	38.3	12.0
		48	334	65.9	17.7	16.5
		72	327	72.8	11.0	16.2
	1 µM	12	277	11.9	39.0	49.1
		24	276	10.9	19.9	69.2
		48	275	5.5	12.0	82.5
		72	266	2.6	9.4	88.0
	10 µM	12	276	11.6	47.5	40.9
		24	273	12.1	24.9	63.0
		48	273	6.6	13.9	79.5
		72	266	4.5	7.9	87.6
Neratinib	0.1 µM	12	121	14.9	57.0	28.1
		24	120	15.0	40.8	44.2
		48	121	20.7	34.7	44.6
		72	116	28.4	22.4	49.1
	1 µM	12	132	12.9	56.8	30.3
		24	131	15.3	37.4	47.3
		48	131	10.7	22.1	67.2
		72	128	7.0	22.7	70.3
	10 µM	12	103	12.6	42.7	44.7
		24	106	8.5	30.2	61.3
		48	105	4.8	8.6	86.7
		72	103	0.0	7.8	92.2
Lapatinib	0.1 µM	12	125	22.4	64.8	12.8
		24	126	42.1	45.2	12.7
		48	124	66.9	19.4	13.7
		72	117	69.2	17.9	12.8
	1 µM	12	126	12.7	65.1	22.2
		24	126	18.3	39.7	42.1
		48	125	18.4	29.6	52.0
		72	126	18.3	25.4	56.3
	10 µM	12	122	11.5	57.4	31.1
		24	122	9.8	27.0	63.1
		48	118	11.9	17.8	70.3
		72	114	11.4	14.9	73.7

Table S5: Proportions of organoids that gained, lost, and maintained mass by treatment condition. (A) Percentage of tracked MCF-7 cell clusters in each condition that gained more than 10% of their initial mass (green), lost more than 10% of their initial mass (black), or maintained a mass within 10% of their initial measured value (gray) at 12, 24, 48, and 72 hours after treatment addition. (B) Percentage of tracked BT-474 cell clusters in each condition that gained more than 10% of their initial mass (green), lost more than 10% of their initial mass (black), or maintained a mass within 10% of their initial measured value (gray) at 12, 24, 48, and 72 hours after treatment addition. Data are plotted in Figure 5A.

		MCF-7		BT-474	
		Viability ± SD	p-value	Viability ± SD	p-value
Vehicle	1% DMSO	1.00 ± 0.22		1.00 ± 0.22	
Staurosporine	0.1 µM	0.50 ± 0.08	<0.0001	0.78 ± 0.09	0.0006
	1 µM	0.17 ± 0.04	<0.0001	0.04 ± 0.01	<0.0001
	10 µM	0.13 ± 0.04	<0.0001	0.01 ± 0.00	<0.0001
Neratinib	0.1 µM	1.02 ± 0.15	0.8463	0.40 ± 0.10	<0.0001
	1 µM	0.92 ± 0.12	0.4623	0.25 ± 0.04	<0.0001
	10 µM	0.08 ± 0.01	<0.0001	0.13 ± 0.05	<0.0001
Lapatinib	0.1 µM	1.00 ± 0.04	0.9452	0.64 ± 0.11	0.0004
	1 µM	1.01 ± 0.05	0.8598	0.38 ± 0.08	<0.0001
	10 µM	0.66 ± 0.08	0.0018	0.26 ± 0.03	<0.0001
	50 µM	0.14 ± 0.04	<0.0001	0.01 ± 0.00	<0.0001

Table S6: Organoid viability analysis by endpoint ATP assay. Comparisons of cell viability measured by ATP assay. P-values are calculated by unpaired t-test with Welch's correction. Data is presented in Figure 5B.

Supplementary Movies

Supplementary Movie 1: MCF-7 organoids treated with vehicle control. Scan the QR code to access the movie or visualize at the following link: https://youtu.be/bUBq-ZChFM0

Supplementary Movie 2: BT-474 organoids treated with vehicle control. Scan the QR code to access the movie or visualize at the following link: https://youtu.be/AzSc8WW5KBA
