# Supplementary material for: Drug screening at single-organoid resolution via bioprinting and interferometry
Source: Nat Commun. 2023 Jun 6;14:3168. doi: 10.1038/s41467-023-38832-8 (PMC10244450; doi:10.1038/s41467-023-38832-8)
Supplement: Supplementary file 3 — Description of Additional Supplementary Files [file 41467_2023_38832_MOESM3_ESM.docx]

**Description of Additional Supplementary Files**

Supplementary Movie 1:

Description: MCF-7 organoids treated with vehicle control.

Supplementary Movie 2:

Description: BT-474 organoids treated with vehicle control.
